# Studying Stem Rust and Leaf Rust Resistances of Self-Fertile Rye Breeding Populations

**DOI:** 10.3390/ijms232213674

**Published:** 2022-11-08

**Authors:** Paul Gruner, Anne Witzke, Kerstin Flath, Jakob Eifler, Brigitta Schmiedchen, Malthe Schmidt, Andres Gordillo, Dörthe Siekmann, Franz Joachim Fromme, Silvia Koch, Hans-Peter Piepho, Thomas Miedaner

**Affiliations:** 1 State Plant Breeding Institute, University of Hohenheim, 70593 Stuttgart, Germany; 2 Julius Kuehn-Institute, Institute for Plant Protection in Field Crops and Grassland, 14532 Kleinmachnow, Germany; 3 KWS LOCHOW GmbH, 29296 Bergen, Germany; 4 HYBRO Saatzucht GmbH & Co. KG, 17291 Schenkenberg, Germany; 5 Biostatistics Unit, Institute of Crop Science, University of Hohenheim, 70593 Stuttgart, Germany

**Keywords:** *Puccinia graminis* f. sp. *Secalis*, *Puccinia recondite*, adult-plant resistance, all-stage resistance, seedling test, QTL mapping, mixed models, cumulative logit models

## Abstract

Stem rust (SR) and leaf rust (LR) are currently the two most important rust diseases of cultivated rye in Central Europe and resistant cultivars promise to prevent yield losses caused by those pathogens. To secure long-lasting resistance, ideally pyramided monogenic resistances and race-nonspecific resistances are applied. To find respective genes, we screened six breeding populations and one testcross population for resistance to artificially inoculated SR and naturally occurring LR in multi-environmental field trials. Five populations were genotyped with a 10K SNP marker chip and one with DArTseq^TM^. In total, ten SR-QTLs were found that caused a reduction of 5–17 percentage points in stem coverage with urediniospores. Four QTLs thereof were mapped to positions of already known SR QTLs. An additional gene at the distal end of chromosome 2R, *Pgs3.1*, that caused a reduction of 40 percentage points SR infection, was validated. One SR-QTL on chromosome 3R, QTL-SR4, was found in three populations linked with the same marker. Further QTLs at similar positions, but from different populations, were also found on chromosomes 1R, 4R, and 6R. For SR, additionally seedling tests were used to separate between adult-plant and all-stage resistances and a statistical method accounting for the ordinal-scaled seedling test data was used to map seedling resistances. However, only *Pgs3.1* could be detected based on seedling test data, even though genetic variance was observed in another population, too. For LR, in three of the populations, two new large-effect loci (*Pr7* and *Pr8*) on chromosomes 1R and 2R were mapped that caused 34 and 21 percentage points reduction in leaf area covered with urediniospores and one new QTL on chromosome 1R causing 9 percentage points reduction.

## 1. Introduction

Rye (*Secale cereale* L.) is a small-grain cereal that is used for food, feed, and bioenergy and was grown on 4.4 million hectares (Mha) in 2020 worldwide with its largest acreage and production in the Russian Federation (1.0 Mha, 2.4 million tonnes; Mt), Poland (0.8 Mha, 2.9 Mt), and Germany (0.6 Mha, 3.5 Mt; [1]). Rye is a cross-pollinating species, and population and hybrid cultivars can be found on the seed market. Hybrid breeding is possible due to self-fertility genes that are necessary for inbred line development and a cytoplasmatic male sterility (CMS) system including restorer genes that is used for large-scale seed production [2]. In 2020, hybrid cultivars (in contrast to population cultivars and synthetics) had a share of about 80% of test fields used for yield and quality assessment in Germany, which approximately represent the general market share throughout Germany [3].

The two most important rust diseases in rye cultivation in Central Europe are stem rust (SR) caused by *Puccinia graminis* f.sp. *secalis* ERIKSS. & HENN. and leaf rust (LR) caused *Puccinia recondita* ROB. Extremely high grain yield losses of 35.8% have been reported for high SR infections in Russia [4]. However, naturally occurring SR infections cannot be observed regularly every year, but when the disease occurs and conditions are favorable (hot, dry, and windy summers), the disease can spread rapidly and cause severe epidemics. Some disease outbreaks in rye were reported in literature, for example in North-eastern Europe, Brazil [5] and South Africa [6], and we can report local disease occurrences on rye in Brandenburg (North-eastern Germany), mainly in dry and hot summers. A yearly updated collection of SR samples from farmers, breeders, and scientific institutes throughout Germany at the Julius Kuehn-Institute (JKI) in Kleinmachnow, Germany, shows that the occurrence has become more common in the last ten years. LR can be observed regularly every year in all rye-growing areas. Losses are generally lower than with SR, but when the disease occurs, up to 39% less yield was reported in rye populations [4]. In rye single crosses, thousand-kernel weight was reduced by naturally occurring LR infections by 14% on average [7]. More general estimates could be extracted from official cultivar registration trials. There, the average yield loss for LR was estimated to be 5.2% and 4.6% for winter hybrid and populations cultivars, respectively [8]. Clear Differences in infection levels between trials with and without fungicide treatment as well as generally high infection levels show a necessity for resistance breeding for LR [8].

Most rust resistances are inherited by single genes and are often active at all plant stages (all-stage resistance, ASR, e.g., [9]). This simplicity is quite appealing for breeding and selection strategies. However, the past has shown that some of those genes, particularly race-specific ASRs, quickly became ineffective with the occurrence of new pathogen races with their respective virulence and it is generally impossible to predict how long a resistance gene will remain effective [10]. The use of diverse and combined (stacked/pyramided) resistances as well as non-race specific resistances, including adult-plant resistances (APRs), could solve this problem and result in long-lasting prevention of rust diseases [10,11]. APR genes do usually have smaller effects and several of them are required to reach near immunity [10]. For SR, ASR and APR were already identified and characterized [4,12,13,14,15], but also genotypes that were resistant only at the seedling stage could be found [13]. However, little was known about marker-linked SR resistance loci in rye and in previous studies three different ASR genes were mapped and a few QTLs were found that were associated with APR [16,17]. Resistance to LR could mainly be attributed to single dominantly acting ASR genes that were active against several pathogen races [18,19] but also breeding lines with APR were found [20]. Several studies were conducted to identify and characterize LR resistances [12,21,22] of which ten genes could be assigned to chromosomes [18,23] and six thereof (*Pr1*–*Pr6*) were linked with molecular markers [23,24,25]. Some of those genes or additional loci may have also been detected by Vendelbo et al. [26].

In this study, we tested new biparental populations in multi-environmental field experiments for SR and LR resistances, and three populations thereof were additionally tested for SR in the seedling stage. The overall aim was to identify new resistance loci in the rye to broaden the portfolio for rye breeders. Beyond this, we want to demonstrate statistical methods for calculating accurate mapping results for both continuous scales of disease severity from field testing and ordinal scales of infection type from seedling tests.

## 2. Results

Six biparental populations, P1-P6, comprising N = 87 (P1–P3), N = 90 (P4, P5) and N = 91 (P6) segregating genotypes (inbred lines) plus their respective parents were analyzed (Table 1, Section 4.1). From P2, additionally a testcross population was developed by crossing each line with a single cross (=tester). Genotypes from all populations were assessed in multi-environmental field trials with artificially inoculated SR and naturally occurring LR. All parents of the populations as well all genotypes from three populations (P3, P5, P6) were additionally tested for SR in the seedling stage. In the following, first, the results for SR resistance (adult and seedling stage) are presented, followed by results for LR field resistance.

### 2.1. Stem Rust

#### 2.1.1. Stem Rust Resistance in Field Tests (Phenotypic Data)

**Figure 1:**
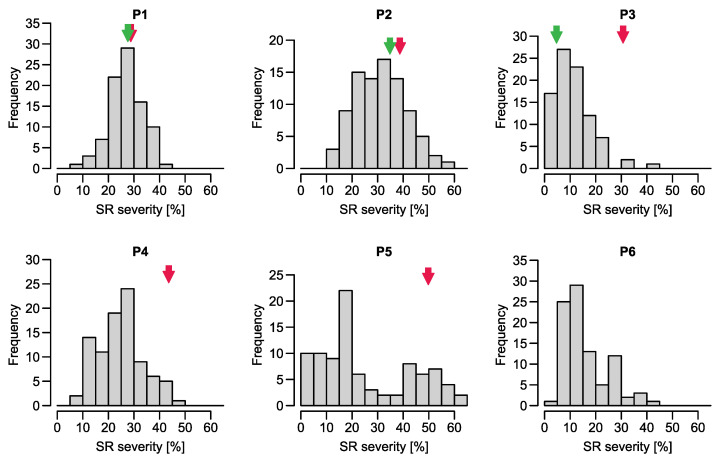
Histograms for populations P1–P6 for stem rust severity. The red and green arrows indicate the value of the susceptible and resistant parent respectively; if not presented, the respective parents of this population were not tested in the field.

Some environments were characterized by low SR infection levels what resulted in low repeatabilities. After removing data from those environments (Table S1), the populations had mean SR severities ranging from 11 to 38% (Table 2). SR severity within the populations ranged between 1.6% and 62.2%. Adjusted means and variance components across environments were calculated for each population separately (Table 3). Heritability estimates ranged between 0.54–0.80, except for P4 and P6, which had a heritability of 0.30 and 0.27, respectively. Those two populations were characterized by high residual error in contrast to the genetic variance (Table 3). Distributions of adjusted SR means appeared centered and rather bell-shaped for P1, P2, and P2TC, left-skewed for P3, P4, and P6, and bimodal for P5 (Figure 1).

**Figure 2:**
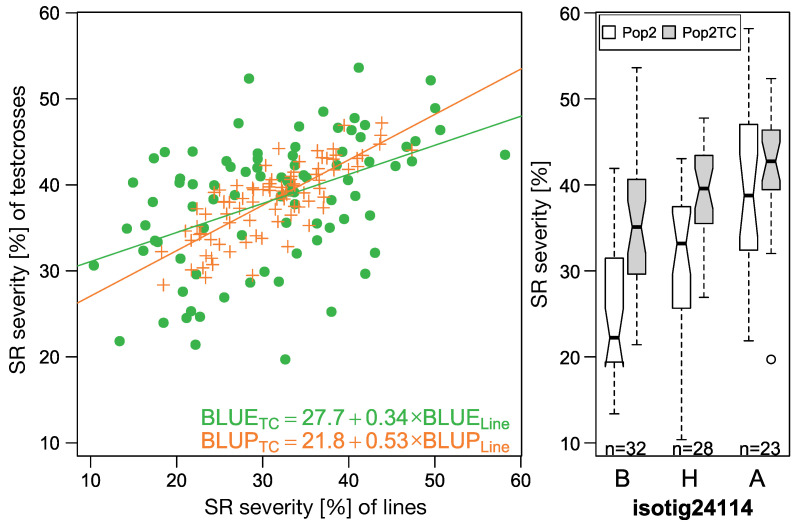
Correlation of line (P2) and testcrosses performance (P2TC) of best linear unbiased estimators (BLUEs) and predictors (BLUPs) for stem rust severity (**left**) and boxplots for the most significant marker associated with SR in this populations (**right**).

Genetic correlation between lines of P2 and the respective test crosses (P2TC) was estimated to be 0.66 for SR (Table S2). The slope of a linear regression of best linear unbiased predictors (BLUPs) of inbred lines on that of testcrosses showed a slope of 0.53, indicating additive inheritance (Figure 2). If best linear unbiased estimators (BLUEs) were considered, the regression slope reduced to 0.34, showing that genetic effects were masked by genotype-environment interaction and residual errors.

#### 2.1.2. Mapping of Stem Rust Resistance in Field Tests

We denominate resistances that clearly showed a qualitative resistance in the field (large effect size and extremely small *p*-value) as major genes and the remaining marker-trait associations as QTLs. If common markers were significant across all populations, we denominate this as the same QTL. Gene and QTL denominations were assigned consecutively to previous studies. We mapped QTLs by using a mixed model with fixed marker effects (single marker regression). To account for heterozygous genotypes, the marker was first fitted in additive coding M_a_ (A, H, B = 0, 1, 2) followed by the same marker with dominant coding M_d_ (A, H, B = 0, 1, 0) what captured any dominance effects of the heterozygous genotypes. Further, fixed marker-environment interactions for again both, M_a_ and M_d_ were fitted. To report marker effects (with standard errors), the difference between the two homozygous states was considered (Hom. effect) what means, in model terms, that M_a_ = 2 and M_d_ = 0. Similarly, also a heterozygous effect (Het. effect) could be calculated when M_a_ = 1 and M_d_ = 1. In a second run, some highly significant markers from a first run were added in the model as cofactors in a second run. However, only in a few cases it resulted in changes in terms of identification of additional or different loci, and only for those cases will we report this cofactor-mapping approach in the following.

**Figure 3:**
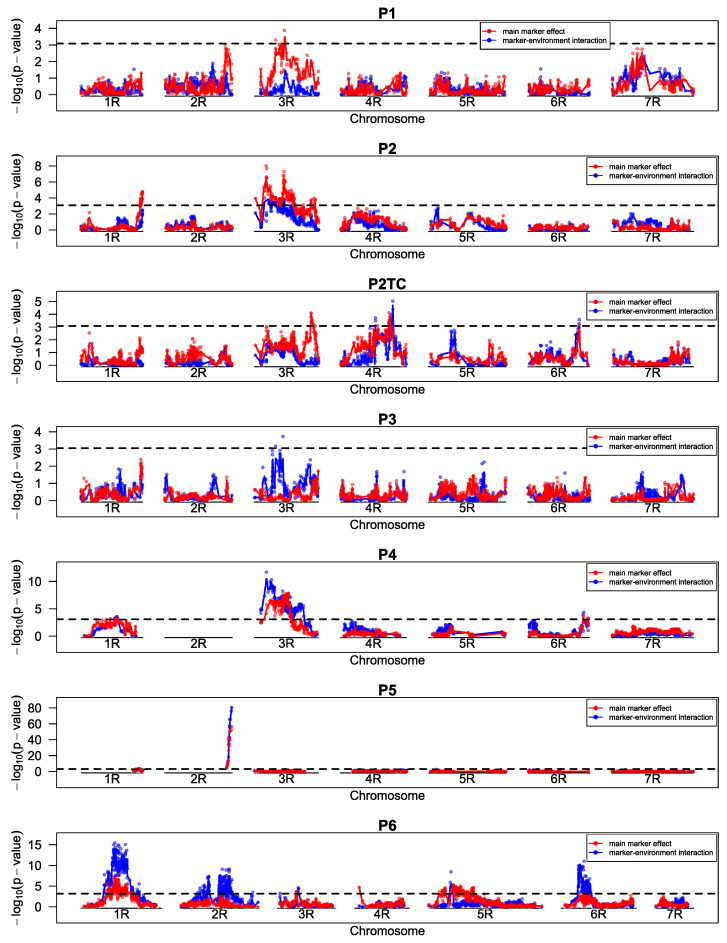
LOD (−log_10_ (*p*-value)) profiles for populations P1–P6 and P2TC for stem rust. *p*-values of the single markers were calculated from a mixed linear model fitting main marker-effects (red) and marker-environment effects (blue) sequentially as fixed effects. Marker order of P1–P5 and P2TC was based on a consensus map from linkage maps of all populations (and populations from previous projects). Marker order of P6 was calculated separately as a different marker system was used. The dashed line shows the global significance threshold (alpha = 0.05) adjusted for multiple testing by the simpleM method.

For SR, we found ten QTL across all populations (except P3) and one major gene in P5 (Table 4, Figure 3). This single resistance gene, *Pgs3.1*, was located on the end of the long arm of 2R and had a large effect of 40.1 percentage points SR reduction and an explained genetic variance (pG) of 0.82. The gene was acting dominantly as we could observe a 3:1 segregation between resistant and susceptible plants within field plots of heterozygous genotypes. In P3, no significant marker-trait association was detected.

Mapped QTLs were characterized by smaller effect sizes ranging between 4.2 and 17.4 percentage points reduction in SR severity (Table 4). The comparison of effect sizes across populations was, however, dependent on different, not fully overlapping test environments. 

One QTL on 3R, QTL-SR4, was found in P1, P2, P2TC, and P4, but in each population a different marker was associated with its largest effect and the largest explained genetic variance. The effects of this QTL ranged between 5.4 (P1) and 12.1 (P4) and pG between 0.24 (P1) and 0.53 (P4). A common marker (isotig33162) was significantly associated with the resistance in all populations and it resulted in only slightly smaller effect sizes and pG (Table S4). Because the testcross population P2TC which was developed from P2 showed about half of the QTL effect (tested in the same environments), an additive gene action could be assumed. On the same chromosome, additional loci were mapped at the top of chromosome 3R in P2 (QTL-SR4.1) and in P6 (QTL-SR12, Table 4) and this region was further characterized by significant marker–environment interaction in P4. Interestingly, for P2TC, another QTL (QTL-SR4.2) was located rather at the end of the chromosome that was not significant in P2.

On chromosome 1R, two QTLs at similar position were identified in P4 (QTL-SR9) and P6 (QTL-SR11), but different marker systems were used for genotyping of both populations and prevented the identification of common markers. In P4, markers of this QTL only slightly passed the global threshold, but when using a marker of QTL-SR4 as cofactor, the *p*-values largely decreased (the LOD increased, Figure S1). Effect sizes for the QTLs were 12.7 and 17.4 and pG was 0.19 and 0.30 in P4 and P6 respectively (Table 4).

Besides those QTLs, more different QTLs were found on 1R (P2), 3R (P6), 4R (P2TC, P6), 5R (P6), 6R (P2TC, P4) and 7R (P1). However, some of them disappeared when fitting a model with other markers as cofactors (P4, P6, Figure S1) or had opposite effects between heterozygous and homozygous states (QTL-SR5, QTL-SR6, QTL-SR7, Table 4).

**Figure 4:**
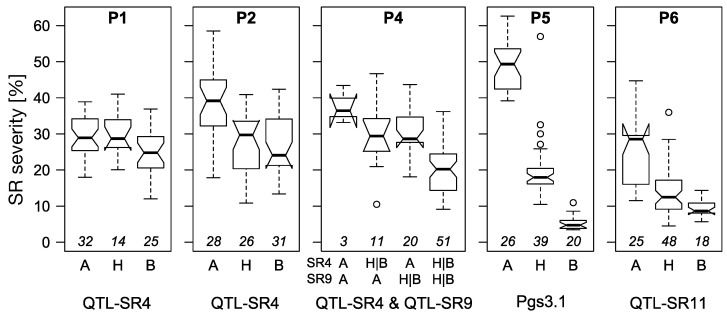
Visualisation of population-wise marker effects of selected stem rust QTLs and *Pgs3.1*. For each population (separate plot), the genotypes were grouped by marker alleles of the associated marker from the respective QTLs/gene and adjusted means across environments were visualized as boxplots. The respective number of genotypes is reported in italics above the bottom line. For P4, the combination of two markers is displayed. For display reasons and for both markers, genotypes with heterozygous allele and homozygous allele B were combined into one group (H|B).

The explained genetic variance increased in all populations by the combination of several QTLs and in all populations two to three QTLs together explained more than 0.39 of the total genetic variance. Interestingly, combinations of the most frequently found QTLs, QTL-SR4, or QTL-SR9 (QTL-SR11) added up nearly additively (or even with an increase in P1) to the total explained genetic variance with other QTLs found in the same populations indicating the absence of epistasis (not all combinations are shown in Table 4 and Table S3). With only one (P2, P6) or two QTLs (P4), high SR reduction could be achieved, but the combination of QTLs did not reach the resistance level caused by *Pgs3.1* (Figure 4).

#### 2.1.3. Seedling Test for Stem-Rust Resistance (Phenotypic Data)

A seedling test was applied for all genotypes from P3, P5 and P6 using isolates that showed different ITs between the parents in a pre-test (Table 5). The other populations were not studied, because the parents and selected genotypes with differing adult-plant resistance from those populations showed no clear differences in SR seedling resistance (Table 5).

**Figure 5:**
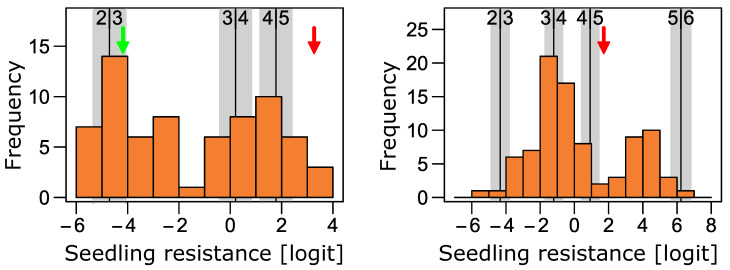
Histograms displaying seedling resistances as adjusted means on a logit scale for genotypes from P3 (**left**) and P5 (**right**). The histograms are based on the model coefficients of the genotypes fitted in a cumulative logit link model (logit scale). The vertical lines display the intercepts (thresholds) for the respective IT groups (2|3, 3|4, 4|5, 5|6) with respective standard errors (grey bars) of the model used to estimate genotype effects. Error display the adjusted means of the resistant (green) and the susceptible parent (red) of the respective populations.

**Figure 6:**
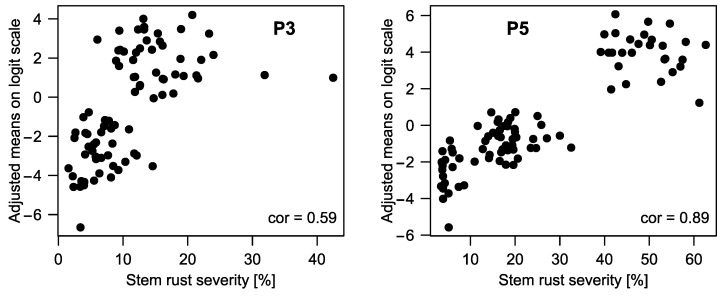
Correlation of genotype means stem rust resistance assessed in the field (*x*-axis) and in the seedling stage (*y*-axis) of P3 (**left**) and P5 (**right**).

In P3, the highest difference between parental lines was between IT 3 and IT 5 (Table 5). When all genotypes of this population were analyzed, most of the variance could be attributed to the genotype effect and no genotype–isolate interaction could be fitted (no model convergence, Table 6). The distribution of the adjusted means clustered into two groups with 42 and 44 genotypes each (Figure 5). Medium correlation (0.59) between adjusted means for seedling and field test could be found (Figure 6), but when fitting the two groups as fixed effects in the phenotypic model, the difference was highly significant (Wald test: *p*-value = 1.5 × 10^−15^) and the group means of field test BLUEs were 6.5% (SE = 2.2) and 15.3% (SE = 2.2). If isolates were considered separately (Figure S2), correlations were weaker and only isolate 43-1 and 173-1 were moderately (0.55 and 0.58) correlated with the results from the field test.

The parents of P5 had the largest IT difference with the largest difference for isolate 3c-3 with IT 1.2 and IT 5.8 for the resistant and susceptible parent, respectively (Table 5). When assessing all genotypes from the population, most of the variance could be attributed to the genotype effect (Table 6) and the correlation between field test and seedling test was high (0.90, Figure 6). Consequently, the genotypes showed a similar (non-normal) distribution in the seedling test as in the field test (Figure 5). Correlation with the field test was also high (0.72–0.93) for all isolates separately (Figure S3), meaning that the ASR was effective against all isolates tested. If we split the phenotypic distribution at the intercept threshold estimate of IT 4 and IT 5 (*α_4_*) for grouping into resistant and susceptible genotypes, the ratios would be 62:28 what does not significantly deviate from a 3:1 ratio according to a Chi-squared test and indicates segregation of a single dominant resistance gene.

For P6, the IT differences between parents were small and only two isolates, 173-1 and 174-1, showed ITs of 4 and 5 for the resistant and the susceptible parent respectively (Table 5). Still, high genetic variance could be found, but this was only based on 14 plants with IT 4 in contrast to 1347 plants with IT 5. This population was thus excluded from further analysis.

#### 2.1.4. Mapping Stem Rust Resistance in The Seedling Stage

**Figure 7:**
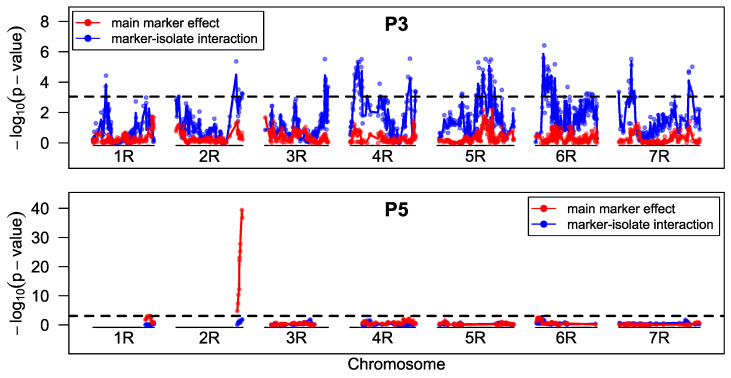
LOD-profiles for mapping of seedling resistance of stem rust in P3 and P5. Marker-wise *p*-values were calculated by comparing likelihood differences (anova) of a cumulative logit link model. Marker order was based on a consensus map from linkage maps of all populations (and populations from previous projects). The dashed line shows the global significance threshold (alpha = 0.05) adjusted for multiple testing by the simpleM method.

In P3, no significant marker-seedling test association could be found and only the marker-isolate interaction was significant and this at almost all loci across the entire genome (Figure 7). When isolates were analyzed separately, either no marker or again almost all markers of the entire genome passed the significance threshold (Figure S4).

For P5, we could find highly significant marker association of the same markers located at 175.2 cM on chromosome 2R of the linkage map from Bauer et al. [27], that were already shown to be associated with the resistance in the adult plant stage (Figure 3 and Figure 7), also when all isolates were analyzed separately (Figure S5). However, the most significant markers of adult-plant and seedling test were different (isotig20303 for field resistance and c26774_197 for seedling test). As seedling test data were analyzed by means of cumulative logit models, the effect sizes of 4.5 for heterozygous and 6.9 for homozygous genotypes were estimated on the logit scale. Those effect sizes are easier to interpret when transferred into the ordinal superiority measure, OSM, in that 96% or 99% of the cases a genotype will have a smaller IT with a heterozygous or homozygous resistance allele, respectively. If this measure is extended for the threshold between IT 4 and IT 5 (OSM1), in more than 99% of the cases genotypes will have an IT below 5, for both heterozygous and homozygous allele states.

### 2.2. Leaf Rust

#### 2.2.1. Leaf Rust Resistance in Field Tests (Phenotypic Data)

**Figure 8:**
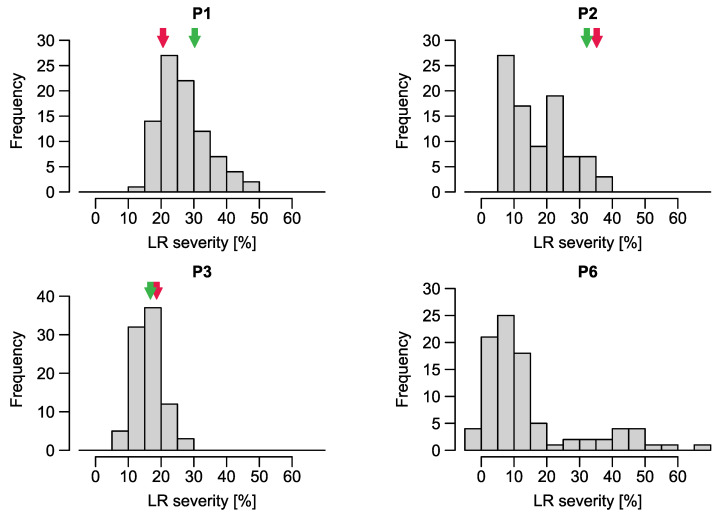
Histograms for populations P1–P3, P6 and P2TC for leaf rust. The red and green arrows indicate the value of the (stem rust) susceptible and resistant parent respectively; if not presented, it was no tested in the field.

The severity of naturally occurring LR and population- and environment-specific repeatabilities were higher than for SR, except for P4 and P5 that were based on crosses of susceptible genotypes and were thus not segregating for LR and for P6 where only one environment was characterized by high repeatability (Table S5). For P1, P2, P2TC and P3, assessments from four to six environments were used (Table 7) resulting in population means between 14 and 27% (Table 7). The range of LR severity within the populations was between 5.6% and 46.0% except for P6 with a maximum of 65.9%, but this could be attributed to the high infection pressure of the single environment in which it was tested. Heritability across environments ranged between 0.58 and 0.83 for P1-P3 and P2TC and only for P1 the variance for the genotype-environment interaction was higher than for the genotype (Table 8). P3 showed a bell-shaped distribution. Distributions of P1, P2, and P2TC were left-skewed and that of P6, bimodal (Figure 8).

**Figure 9:**
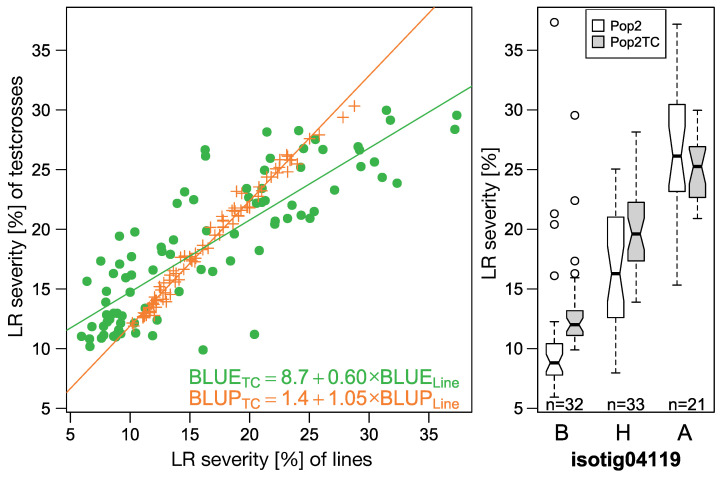
Correlation of line (P2) and testcrosses performance (P2TC) of best linear unbiased estimators (BLUEs) and predictors (BLUPs) for leaf rust severity (**left**) and boxplots for the most significant marker associated with LR in this populations (**right**).

Genetic correlation between lines of P2 and the respective test crosses (P2TC) was estimated to be 0.97 (Table S1) and the linear regression of BLUPs of inbred lines on that of testcrosses showed a slope of 1.05, indicating dominant resistance (Figure 9). As with SR, regression slopes of BLUEs were again lower than for BLUPs (0.60).

#### 2.2.2. Mapping of Leaf Rust Resistance in Field Tests

**Figure 10:**
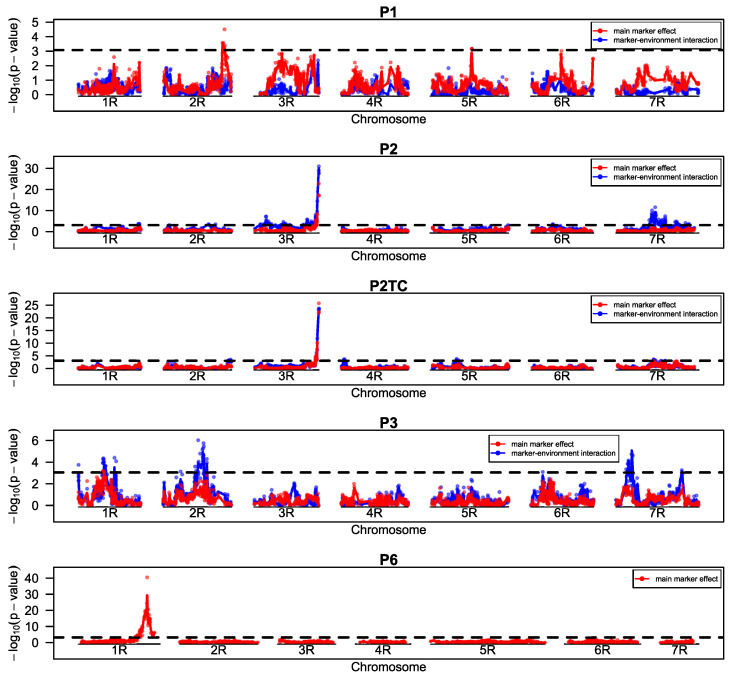
LOD (−log_10_ (*p*-value)) profiles for populations P1–P3, P6 and P2TC for leaf rust. *p*-values of the single markers were calculated from a mixed linear model fitting main marker-effects (red) and marker-environment effects (blue) sequentially as fixed effects. Marker order of P2–P5 and P2TC was based on a consensus map from linkage maps of all populations (and populations from previous projects). Marker order of P6 was calculated separately as a different marker system was used. The dashed line shows the global significance threshold (alpha = 0.05) adjusted for multiple testing by the simpleM method. Mapping in P6 was based on data from a single location only.

For LR, two genes were found, *Pr8* on 1R and *Pr7* on 3R (Figure 10, Figure S6). *Pr7* was found in P2 and P2TC and had an effect size of 20.7 and 17.6 percentage points in LR reduction and pG of 0.62 and 0.65 respectively (Table 9, Table S6). Even though the effect sizes of P2TC were slightly smaller than that of the line population, a dominant gene action could be inferred already from the genetic correlation between the inbred lines and the testcrosses (Figure 9). The detection of *Pr8* on chromosome 1R in P6 was based on phenotypic data from HOH in 2020 only. Based on these data, the effect was estimated to be 33.7 and pG 0.74 (Table 9). Only one QTL for LR, QTL-LR3, was found in P1 on chromosome 2R with effect size and pG of 8.6 and 0.28, respectively.

## 3. Discussion

### 3.1. Challenges of Using Rye Breeding Material for Mapping

A big challenge is the naturally cross-pollination character of rye and inbred lines can only be produced due to a self-fertility gene. Each self-pollination, requires isolation bags and results in only a few grams of seed per bag. Moreover, inbred lines are characterized by inbreeding depression that is characterized by loss of vitality. The production of double haploid lines has not been successful yet in elite rye breeding material. This all represents a big difference to self-pollinating species, where inbred lines can be produced with low workload and in almost unlimited population sizes and seed quantities.

This project was based on a private-public partnership and thus material from the respective company could only be tested on company-owned sites and stations of the scientific partners. This limited the comparison of absolute values of genotype means across different material. However, as the purpose of this study was to identify new resistance loci within each population, the calculation of means across environments for each population separately suffices. To further reduce the error for mapping, the genotypes from each single population were always grown together in randomized experiments at all locations.

Special attention was paid to heterozygous genotypes that were in rather early inbreeding generation. To conduct replicated experiments in different environments, we tested genotypes in single rows (plots). Plants of a heterozygous genotype with a single dominant gene grown in a single plot (DNA sampling from seed bulk), were segregating within the plot and as an average score per plot was given, a fully dominant gene (1:3 segregation within the plot) could never reach the same resistance level of the (homozygously) resistant parent. This must be considered when interpreting effects of heterozygous genotypes. We discussed this already in detail in our previous study [16] and tried to adjust the marker coding for QTL mapping there. In this study, we used another approach by fitting an additional dominance effect for each marker. This allowed a more flexible estimate for any kind of dominance effects. An additional consequence of this within plot segregation of heterozygous genotypes was the potential of additional errors, with just slightly different allele ratios caused by the limited number of plants per plot. Actually, we observed very high standard errors of our QTL effect estimates (Table 4 and Table 9). However, we considered this experimental side effect of less importance than the need for multi-environmental field trials. This segregation problem of course also affected the seedling test in similar fashion and the dominance effect estimated across single plants from the same genotype was not as high as would had been expected for a full dominance effect on single plant basis.

Another experimental challenge was the choice of appropriate population size. Grains for multi-environmental field trials were limited due to aforementioned reasons and to broaden a breeder’s portfolio of resistance sources, it was reasonable to test several populations with small population sizes. Further, minimum population size could also be calculated from statistical theory, e.g., in Falconer and Mackay [28] a formula from Sokal and Rohlfs [29] is used: $n\;\geq 2\;{\left(z_{\alpha }+z_{2\beta }\right)}^{2}/{\left(\frac{\delta }{\sigma_{W}}\right)}^{2}$. According to this formula, the size of each marker allele group depends on the smallest relevant difference *δ* between marker classes, its standard deviation *σ_W_*, and the quantiles of the normal distribution *z* for the acceptable error rate of false positives (*α*) and false negatives (*β*). If both, *α* and *β*, are set to 0.05, *δ* to 10, and based on standard error estimates between 1.7 and 6.7 from previous studies [16], that would be equal to *σ_W_* between 10 and 40, n would be between 26 and 400. Thus, our chosen population sizes of about 90 (*n* = 45) was within this rage, but it becomes clear that *σ_W_* is the crucial parameter for planning of experiments and the low heritability for SR in some populations resulted in very large standard errors (Table 4). The formula used to calculate the sample size was based on differences detected by a *t*-test. A thorough review on approaches to determine sample sizes can be found in [30].

Additionally, we considered it as a proof of methodology that loci across populations were found at similar chromosomal positions and loci, such as *Pgs3.1*, QTL-SR9/QTL-SR11, and QTL-SR8/QTL-SR10, were close to or overlapping loci found previously (*Pgs3*, QTL-SR1, QTL-SR3 in [16,17]).

### 3.2. Resistance to Stem Rust

Because SR does not yet occur regularly at all locations, plots were artificially inoculated. This, however, was no guarantee for high SR severity and in some environments, severities were very low. This resulted in low repeatability estimates and data from the respective environments was dropped resulting in less test environments for analysis across environments (Table S1). We attributed the low SR severity to unfavorable weather conditions, especially in 2020 when the spring and summer was wet and cold. In 2017 and 2018, we performed similar experiments for SR resistance with other breeding material in almost the same locations and could achieve generally higher infection levels and higher repeatabilities for the single locations [16]. The modelling of fixed marker-environment interaction (instead of random) in the mapping procedure should further help to additionally track those environmental differences as significant marker-environment effects in QTL mapping and prevent that the actual marker effect would be lost in a large random marker-environment interaction.

Unexpectedly, we found only one single ASR gene for SR in six populations. In wheat, most SR resistance genes are ASR genes and to the best of our knowledge already *Sr* gene number 62 has been described [31], that was like many other before derived from introgressions of other cereals or grass species. Reasons why, in contrast to this study on rye, only a few QTLs for APR for SR in wheat were known, may be that in wheat studies most of the testing was done in the seedling stage or that the cross-pollinating character of rye generally results in a larger resistance gene diversity and during evolution, domestication and breeding of rye, APR QTLs accumulated as consequence of higher plant fitness. Overall, we found 10 SR-QTLs with effect sizes ranging from 4 to 17 percentage points SR reduction. Three pairs of SR-QTLs were mapped at similar chromosomal regions (QTL-SR9/QTL-SR11, QTL-SR7/QTL-SR13, QTL-SR8/QTL-SR10). Interestingly, and a bit unfortunately for breeders, some QTLs for SR resistance, e.g., QTL-SR4 or QTL-SR9/QTL-SR11, were located in centromeric regions that were generally characterized by low recombination frequency [32]. Similar results have been observed for barley [33]. There, most of those race-nonspecific genes were even active against several pathogens making those genes more appealing for breeders. In our material, no QTL could be proven to be active against SR and LR simultaneously.

To the best of our knowledge, no other marker-linked resistance loci for SR resistance in rye has been published, except in our previous studies [16,17]. QTLs and genes with overlapping positions or close by previously identified loci were *Pgs3*.1 close to *Pgs3*, QTL-SR9/QTL-SR11 close to QTL-SR1 as well as QTL-SR8/QTL-SR10 and QTL-SR3. However, the same marker position is no proof for having the same gene. Moreover, our definition of two QTLs in different populations being the same QTL if a common marker could be used to detect it is only a further argument for it and no final proof. Common markers were most probably not detecting the causal mutation in the respective gene. Even though no rye-SR genes were found, material and markers were overlapping with other studies. The population Gator, which was the resistance donor of *Pgs3.1,* has been studied by Tan et al. [13,14] and they also found a single dominant gene (*SrC*) that could be the same as *Pgs3.1.* A wheat-SR gene, *Sr59,* which is located on a rye translocation in wheat, was associated with three markers from a rye SNP chip [34,35]. One of them (c20194_115) could be referenced on the linkage map of Bauer et al. [27] at 175.2 cM, which is close to *Pgs3.1*. The population Elbon, which was the resistance donor of P4, was studied by Tan et al. [13,14], too. However, for this population, the results did not agree well with ours. When studying the response to f. sp. *secalis*, they found genes in the seedling stage that were not active in the adult-plant stage, and when studying the response to f. sp. *tritici,* they found two dominant genes active in seedling and adult plant stage.

By identifying field resistance loci in the rye genome, a seedling test was necessary to distinguish ASR from APR. However, only *Pgs3.1* from P5 was segregating in both, seedling and adult-plant stage. This could be shown by correlating adjusted means from seedling and field test, but also by mapping the same resistance locus based on both tests. The difference between susceptible and resistant genotypes was already observed when parents of this population were tested in a pre-test for seedling resistance. Accordingly, among parents from all populations, the parents of P5 had the largest IT differences.

As smaller differences between parents from other populations (P3, P6) could also be observed in the seedling test (Table 5), the question concerned how this difference in resistance was related to field resistance and whether responsible gene loci could be found. P3 was studied in detail, because its parents had different ITs in the seedling stage and high genetic variance was also found when analyzing the offspring. Based on a correlation of 0.59 between adjusted means from field and seedling experiments, it was concluded that some genes/QTL must be different between seedling and adult-plant stage, but also some common genes must be given. The two clusters observed in the seedling stage (Figure 5 and Figure 6) were also significantly different based on field data. Surprisingly, no resistance locus could be found in this population (Figure 3 and Figure 7). We repeated the seedling test for all genotypes with a detached leaf-segment test but, again, no significant marker–trait association was found.

Results pointing in the similar direction were reported by Tan et al. [13] who discovered genes based on phenotypic segregation causing phenotypic differences in the seedling stage but not at the adult-plant stage. Johnson [36] studied environmental factors affecting the infection level in SR seedling tests of wheat and discovered for some isolates and genotypes a highly environment-dependent resistance reaction, which was termed X-type reaction, and with similar definition, this X-type reaction could also be found in the work of Stakman [37]. In our experiments, an X-type would be expressed as differences between plants of a single genotype. As we fitted a statistical model to analyze our data, the presence of X-types would be expressed by low genetic variance and high variances of experimental factors instead, but this was not observed (Table 6).

### 3.3. Resistance to Leaf Rust

LR infections are occurring naturally in all environments. LR is much more adapted to the climate in Central Europe and in contrast to SR the urediniospores can overwinter on the crop and thus easily boost the disease as soon as the conditions are favorable. However, the consequence of natural inoculum is that there may be different pathotypes of the fungus at different locations. Again, the mapping statistics was adjusted for such cases by modelling a fixed effect for marker-environment interaction. One LR resistance gene, *Pr7*, was active in all locations and did not show significant marker-environment interaction (Figure 10, Table 7). The other identified resistance gene, *Pr8*, was only segregating in one environment and should be again tested in more environments.

For LR resistance in rye, to the best of our knowledge only ten chromosome-associated and thereof six marker-associated genes have been reported [18,23]. No marker-linked genes were reported on chromosome 3R and thus the LR resistances detected in P2 and P2TC was assigned with the tentative name *Pr7*. On chromosome 1R, several LR resistances have been found [25,26,38]. However, it is difficult to compare the previously found genes with ours. According to chromosomal location, the most reasonable genes would be *Pr3*, *Pr4,* or *Pr5* [25], which were all located between the two markers *Xscm1* [39] and *Xps162* [40]. The BLAST of the respective sequences resulted in alignments at 137,570,945bp and 629,653,045bp in the Lo7 reference genome [32], respectively, and our best marker for the LR gene on 1R aligned at 713,072,950bp, outside the defined interval. Interestingly, the associated marker in Rakoczy-Trojanowska et al. [38], *3363612* (pers. commun. Rakoczy-Trojanowska), mapped on chromosome 2R (not on 1R) in the Lo7 reference genome. The marker AX-99805135 associated with leaf-rust resistance in Vendelbo et al. [26] was aligned at 625.54 MB in the Lo7 reference genome and was thus close to ours. For this, however, also no previously mentioned gene denomination was proposed, and thus we assigned the LR resistance found in P6 to a new locus *Pr8*.

### 3.4. Breeding Rust-Resistant Hybrid Rye

Hybrid cultivars are usually based on three- or four-way hybrids with different genetical composition (CMS vs. restorer pool). This would allow breeding strategies with different resistances in both breeding pools and stacking of dominant genes in the final hybrid. We have shown for *Pr7* and *Pgs3.1* that the resistances for LR and SR act dominantly. Similar results as well as additional genes have been reported elsewhere [17,18] and combinations of all could be used as stacked resistance genes. The use of the detected APR QTLs would require more breeding effort, because effect sizes were smaller (approximately by factor 2–3, Figure 4) and thus several QTLs must be added up to result in a cultivar with high resistance level. In our material, the combination of a single or two QTLs already resulted in very resistant genotypes (Figure 4). Unfortunately for breeding, the APR resistance QTLs were not acting dominantly, so that in the case of rye hybrids, the QTLs must be introgressed into both pools to get the full effect. However, it is not known which level of resistance would be necessary to prevent the cultivars from yield losses caused by SR infection and small effect genes could already slow down rust infections until harvest, which is in Central Europe generally a bit earlier than the harvest of winter wheat.

Our study shows that there is a large potential to further optimize rye cultivars in terms of resistance. If our results presented here could be transferred into diagnostic marker sequences in future studies, breeders would be well equipped and not restricted to intensive field testing anymore.

## 4. Material and Methods

### 4.1. Mapping Populations

Six biparental populations, P1–P6, comprising inbred lines with N = 87 (P1–P3), N = 90 (P4, P5) and N = 91 (P6) segregating genotypes plus their respective parents were analyzed (Table 1). Resistance donors were either breeding material from the companies KWS LOCHOW GmbH (KWL), Bergen, Germany, and HYBRO Saatzucht GmbH & Co. KG, Schenkenberg, Germany, or genetic resources from the N. I. Vavilov All-Russian Institute of Plant Genetic Resources, Saint Petersburg, Russia (VIR819) or the US-American varieties Elbon and Gator, that were stored and multiplied at University of Hohenheim (UHOH), Stuttgart, Germany. The populations P4 and P5 were based on backcrosses with the susceptible inbred line L301-N. The original resistance donor was a single plant from the respective population and could not maintained due to its self-incompatibility. Susceptible parents were generally self-fertile inbred lines and inbreeding generations of the populations ranged from F_2:3_ (or BC_1_F_2:3_) to F_2:5_. From P2, additionally a testcross population was developed by crossing every line with a single-cross (=tester). The offspring were assessed in this study in the same environments as the line population.

### 4.2. Field Test

Two-year field trials with scorings in 2019 and 2020 (sowing in autumn 2018 and 2019) were conducted in Germany in Stuttgart-Hohenheim (HOH), Berlin-Dahlem (DAH), Petkus near Baruth/Mark (PET), Wohlde near Bergen (WOH), Kleptow near Prenzlau (KLE) and Wulfsode near Wriedel (WUL). Not all material was tested in all locations or environments or was excluded due to low disease pressure, finally resulting in 2 to 6 environments (year-location combinations) for each population, except for P6 and LR where only one environment had sufficient disease pressure (Tables S1 and S5). P4 and P5 were based on crosses between two LR susceptible lines and were thus not considered for this trait. At each location, inbred lines were grown in single rows (plots) with 1 to 1.5 m length (30–60 grains/row) separated by susceptible (spreader) lines and randomized according to an alpha design with two replicates.

Inoculation and scoring followed the same procedure as reported in Gruner et al. (2020). Spores from three different SR isolates were used for inoculation. The virulence pattern on previously developed differential lines [41] is reported in Table S7. The spores were produced on seedlings of the susceptible rye cultivar “Palazzo”. The field trials were inoculated two to three times in the interval from mid heading of plants until end of flowering (BBCH 55–65) with 5–10 days in between by means of an urediniospore-agar suspension (0.1% agar, 120 mg of spores per 100 m^2^) using a spinning disk sprayer (Micron Ulva, Bromyard Industrial Estate, Bromyard, UK). Starting with the first visually distinguishable symptoms (beginning/mid of kernel ripening, BBCH 80–84), the percentage of stem surface between the second leaf from top (F-1) and the node above covered with uredinia was visually assessed (0–100%). For a single score, stems from all plants in the plot were considered resulting in an average score for each plot. Rating was repeated three times at intervals of about one week. For LR, field trials were not artificially inoculated, but natural occurring infections were assessed plotwise as percentage of leaf surface of the F-1 leaf covered with uredinia (0–100%).

### 4.3. Seedling Test (ST) for Stem Rust Resistance

A seedling test (ST) for SR resistance was conducted in 2020 at the Julius-Kuehn Institute in Kleinmachnow, Germany. A pre-test of the parents showed that only the parental lines of P3, P5, and P6 were differentiating with the used isolates. Thus, we restricted the ST to those populations. The plants were grown in multipot-trays (35 pots with 4 × 4 cm). All grains from a single genotype were sown into one pot and all genotypes were sown in the order of ascending genotype IDs in the multipot-trays, thus resulting in 35 genotypes per tray. Plants were sprayed with an agar-uredospore suspension (0.1% agar and 0.25 mg spores per plant) in the 3- to 4-leaf state by means of a thin layer chromatography sprayer (with a small compressor). In total, six single-pustule isolates (isolates initially derived from a single pustule) showing different virulence/avirulence patterns on 15 differential lines (Table S7) were used, but not all were tested on all populations. Five isolates (3c-3, 3h-3, 6-1, 11-4, 43-1) were used previously in Gruner et al. [16], three (3c-3, 3h-3, 43-1) in Gruner et al. [17] and three isolates (3c-3, 3h-3, 43-1) were also used for field inoculation. After inoculation, the plants were kept in the dark for 24 h and 100% humidity and were then transferred into a greenhouse (16 h light/8 h dark) where humidity was also kept at 100% until first symptoms of chlorosis were visible. The plants were scored 14 days after inoculation. All leaves from a single plant were assessed separately, but as the plants continued to grow from the time point of inoculation until scoring, only the leaf with the maximum score was considered for analysis. On average, 5–8 plants per genotype were inoculated, but some plants did not survive until scoring or developed no clear symptoms such that sometimes fewer observations were available (Table S8).

The following rating scheme used to assess the infection types (IT) was adapted from the work of Stakman et al. [37] (in brackets):

(0): no symptoms
(0;): hypersensitive flecks
(1): very small pustules surrounded by necrosis
(2): small- to medium-sized pustules surrounded with chlorosis or necrosis
(3): medium-sized pustules with or without chlorosis
(4): large pustules without chlorosis

### 4.4. Phenotypic Data Analysis—Field Trials

For each single assessment of each trait and for all populations in all environments separately, a basic mixed linear model (1) $y_{ilk}=\mu +g_{i}+r_{l}+b_{lk}+e_{ilk}$ was fitted to calculate repeatability (=single-location heritability), with $y_{ilk}$, the response modelled by the intercept *μ*, the random effect of the *i*th genotype *g_i_*, the effect of the *l*th replicate *r_l_*, the effect of the *k*th block nested within the *l*th replicate *b_lk_* and the error term *e_ilk_*. Repeatability (and heritability later on for model 2) could be calculated by $Rep=\frac{\sigma_{g}^{2}}{\sigma_{g}^{2}+\bar{v}/2}$, where $\sigma_{g}^{2}$ is the genetic variance and $\bar{v}$ the mean variance of a difference of two best linear unbiased estimators (BLUEs) when fitting genotype as fixed effect [42]. Repeatability was used to select the best assessment (=highest repeatability) in case traits were scored several times and to generally discard data from environments with very low repeatability for the final analysis across environments. Data were discarded especially in the case of SR, where, despite artificial inoculation, disease severity was low in some environments finally resulting in two (P6), three (P2TC, P4, P5) and four (P1, P2, P3) environments per population with environment-wise repeatability between 0.44 and 0.98 (Table S1). For LR, data from four (P1), five (P3), and six (P2, P2TC) environments were used, except for P4 and P5 that were based on two LR-susceptible parents and were thus not considered, and P6 which only showed high LR infections and high repeatability (0.90) in one environment (Table S5).

To fit a population-wise model across all environments (model 2), the basic model (1) was extended by effects for the *l*th year-location combination (=environment, *s*) and the respective interaction with genotype *gs*. The model (2) can be written as $y_{ijklm}=\mu +g_{i}+s_{j}+r_{jl}+b_{jkl}+{\left(gs\right)}_{ij}+e_{ijklm}$ Indices again represent the nesting structure. For the replicate and block effects as well as for the residual error we defined environment-specific (heterogeneous) variances. All effects were fitted as random, except the genotype when calculating BLUEs.

Special attention was paid to the testcross population P2TC, which was developed by crossing each single line from population P2 with the same tester genotype. Consequently, both populations shared a single allele for each marker and this genetic correlation could be modelled by extending model (2) into a bivariate form (=model (2a)) and by estimating covariance (unstructured variance-covariance and general correlation) for the genotype effect and the respective genotype-environment interaction. Separate effects (diagonal variance-covariance) were fitted for the environment. The replicate and block effects as well as the residuals were again fitted with heterogenous variances between the population-environment levels. All models were fitted using ASReml 4.0 [43] within RStudio [44].

### 4.5. Phenotypic Analysis—Seedling Test

Because the ITs are ordinal data, cumulative mixed logit models (clmm) were used to calculate genotype means and estimate variance components for data from the seedling test. Model (a) was fitted by $logit\left[P\left(Y_{ikmn}\leq j\right)\right]=\alpha_{j}+p_{i}+g_{k}+{\left(pg\right)}_{ik}+e_{m}+f_{n}$ and gives the log odds (logit) of the response $Y_{ikmn}$ to fall in category j (*j* = 1, …, 6) or below by the effect *p* for the *i*th isolate, the effect *g* for the *k*th genotype, the genotype-isolate interaction (*pg*)*_ik_,* and the two crossed effects $e_{m}+f_{n}$ for the *m*th multipot tray *e* and the *n*th position *f* on the multipot tray. For estimating variances, all effects were fitted as random. For estimating model coefficients for the genotypes, the isolate and the genotype were fitted as fixed factors. For P3, it was not possible to fit a genotype-isolate interaction (no model convergence) and for P6 it was not possible to estimate standard errors for the coefficients (singularity in Hessian matrix) and thus the isolate effect (only two isolates) was modelled as random to estimate variance of the other factors. The cumulative mixed logit models were fitted with the clmm-function of the R package ordinal [44,45]. All models were fitted with the Laplace approximation.

### 4.6. Marker Analysis

Two different marker systems were used. P1–P5 were analyzed with a custom 10k Infinium iSelect SNP chip that was proprietary to KWS SAAT SE & Co. KGaA, Einbeck, Germany. Several markers from this chip were overlapping with the 5 k-SNP assay of Martis et al. [34] and the 600 k-SNP assay of Bauer et al. [27]. P6 was analyzed using the genotyping-by-sequencing (GBS)-based DArTseq^TM^ technology (Diversity Arrays Technology Pty Ltd., University of Canberra, Australia) and filtered SNP markers were used in the analysis. For comparison with our maps as well as previously published genetic maps, Diversity Arrays Technology provided the position for numerous SNP markers on scaffolds of the rye genome draft of Bauer et al. [27]. DNA was extracted from a seed sample of six grains using respective protocols of the companies. Marker sequences are proprietary to the sequencing companies. However, to allow comparisons with other studies, physical marker positions in base pairs (bp) in the Lo7 reference genome [32] of the respective markers were reported ± 1 kbp accurately (Table S9).

Marker datasets were transformed into ABH format (A = allele of susceptible parent, B = allele of resistant parent, H = heterozygous) for each population separately and filtered according to the following criteria: Markers with a call rate (CR) below 0.95 and without three allele states were dropped and genotypes with minor allele frequency (A or B allele) < 0.1 and CR < 0.8 across all markers were dropped as well. Additionally, markers of each population were correlated and from groups with redundant markers (correlation > 0.99) all except one with highest CR were dropped for the mapping procedure. This finally resulted in marker datasets with 82–91 genotypes and between 299 and 1025 non-redundant SNP markers for the SNP chip method and 3352 non-redundant SNP markers for the DArTseq^TM^ method. More details and descriptive marker statistics can be found in Table S10.

### 4.7. Linkage Map Construction

The mstmap-function from the ASmap package [46] was used to construct population specific linkage maps. Markers overlapping with the map constructed by Bauer et al. [27] were used to assign linkage groups and orientation (Figures S7–S12). The resulting map of P6 based on DArTseq^TM^ markers was relatively large. To improve this, we polished the data using the ABHgenotypeR package [47]. For this purpose, a first map was constructed with ASmap and this order was then used to import the marker data into the ABH format of the package. We then used the functions correctUndercalledHets with maxHapLength = 5 and correctStretches with maxHapLength = 5 to polish the dataset. The resulting new marker dataset was again used for linkage map construction with ASmap. Map length before and after polishing can be found in Table S11.

### 4.8. Mapping Procedure—Field Data

For QTL mapping, each marker of each population was regressed on the phenotypic data by extending model (2) with fixed effects for the *zth* markers *m*. Each marker (alleles A, H, B) was coded additively (a) with levels 0, 1, 2 (alleles A, H, B) and additionally dominantly (d) with levels 0, 1, 0 (alleles A, H, B) to capture the deviation of heterozygous genotypes from the additive gene action. These two fixed regression coefficients *m*_1_ and *m*_2_ were followed by marker–environment interaction for again both additive and dominant marker coding (*m*_3_ and *m*_4_). The model (3) can be extended as $y_{ijklm}=\mu +m_{1}a_{zi}+m_{2}d_{zi}+m_{3}{\left(as\right)}_{zij}+m_{4}{\left(ds\right)}_{zij}+g_{i}+s_{j}+r_{jl}+b_{jkl}+{\left(gs\right)}_{ij}+e_{ijklm}$, where now *g_i_* and ${\left(gs\right)}_{ij}$ model the residual genetic and genotype-environmental variation not accounted for by the markers. The *p*-values could be derived from the (incremental) Wald-statistics of the fixed effects, and it must be noted that due to this sequential fit and incremental Wald-statistics, *p*-values of the 2nd, 3rd, and 4th effect were dependent on the preceding effects. In ASreml, an approximate F-statistic is reported that is calculated by dividing the Wald statistics by the numerator degrees of freedom. In our case of (marker) regression analysis, there is only one numerator degree of freedom for each regressor and thus the F-statistic could be directly compared with a chi-squared distribution. For visualization (LOD score/Manhattan plot), we added up the Wald-type chi squared statistics of additive and dominant effects and respective environment interactions and calculated *p*-values for marker-main effect and marker-environment interaction. Marker effects were predicted (ASReml predict function) for the heterozygous state (*a* = 1, *d =* 1) and for the homozygous state (*a* = 2, *d* = 0). The explained genetic variance (pG) was calculated directly from the difference of estimated genetic variance in model (3) and model (2) divided by the estimated genetic variance in model (2) and is reported as proportion throughout the paper.

QTL with very large effects and high explained genetic variance, which are typical characteristics for resistance genes, were termed as genes, but of course also all other QTLs could be assumed to harbour genes causing the phenotypic differences, but with smaller effect sizes. As effect size of a gene/QTL, we consider the difference between the homozygous states (A and B) for the respective marker. Heterozygous marker allele states could be used to interpret the dominance effect of the genes. However, the phenotype in the field was assessed as average score for a single row with several plants and consequently the plants of a dominant gene in heterozygous state (DNA was also extracted from a bulked seed sample) would segregate in a 1:3 ratio resulting in 75% (and not 100%) of the (homozygous) gene effect. If the phenotypic differences between resistant and susceptible plants were really large (like for SR in P5), this segregation could be clearly observed in the field. Accordingly, the genotype effect of an additive gene would result in 50% of the (homozygous) effect size.

After screening the genome for marker-trait associations, markers were plotted in the order of the linkage map and from the most significant loci, single markers were chosen that were fitted also with additive and dominant coding and with marker-environment interaction as cofactors in model (2). We constructed a pairwise recombination matrix for all markers and excluded markers from the cofactor model that had a recombination rate of less than 20 cM in each marker fit. *p*-values of this second genome scan were again extracted from Wald statistics.

### 4.9. Mapping Procedure—Seedling Data

To assess marker-trait associations for the seedling test data, the phenotypic model (a) was extended by fixed effects for each marker separately. The markers were fitted sequentially as additive (a, A, H, B = 0, 1, 2) and dominant (d, A, H, B = 0, 1, 0) effects and with respective isolate-marker interactions so that the model (c) for the marker tests can be written as $logit\left[P\left(Y_{ikl}\leq j\right)\right]=\alpha_{j}+\beta_{1}a_{kz}+\beta_{2}d_{kz}+\beta_{3}ap_{kzi}+\beta_{4}dp_{kzi}+p_{i}+g_{k}+{\left(pg\right)}_{ik}+d_{l}$. To compute a *p*-value of additive and dominant marker effects together (without isolate interaction), a further model (b) was fitted, i.e.,$\;logit\left[P\left(Y_{ikl}\leq j\right)\right]=\alpha_{j}+\beta_{1}a_{kz}+\beta_{2}d_{kz}\;p_{i}+g_{k}+{\left(pg\right)}_{ik}+d_{l}$. The *p*-value of a log-likelihood test (anova function; R Core Team, 2019) of model (a) and (b) was considered for marker-main effects and *p*-value of loglikelihood differences of model (b) and (c) were used to assess marker-isolate interaction. Additionally, models (without isolate interactions) were fitted for each isolate separately, however for isolates 43-1 and 173-1 in P3 and 3c-3 in P5 it was not possible to fit a tray effect. Effect estimates of the markers were extracted from the model coefficients (*β_1_*, *β_2_*, model c) and transferred into estimates for the heterozygous phase B_het_ (a = 1, d = 1) and for the homozygous phase B_hom_ (a = 2, d = 0). Because those measures were difficult to interpret, they were transformed into into the ordinal superiority measure (OSM; [48]). The OSM was calculated as $OSM\approx \exp\left(\frac{B}{\sqrt{2}}\right)/\left(1+\exp\left(\frac{B}{\sqrt{2}}\right)\right)$ and gives the probability [0, 1] that the infection type is lower with the respective resistant (homozygous or heterozygous) allele state compared to the susceptible one. We extended this measure to a defined threshold (*α_4_*, threshold between IT4 and IT5) that was sometimes considered as border between resistant and susceptible reaction [37] and thus gives the probability to fall into the resistant category given the respective allele state. The extended measure OSM1 was calculated by $OSM1\approx \exp\left(\frac{\alpha_{4}+B}{\sqrt{2}}\right)/\left(1+\exp\left(\frac{\alpha_{4}+B}{\sqrt{2}}\right)\right)$.

### 4.10. Significance Threshold

The global significance threshold was adjusted to correct for multiple (marker-wise) testing using the simpleM method proposed by Gao et al. [49]. Instead of correcting naively for all tests (= number of markers) with Bonferroni correction, an effective number of markers q_eff_ was calculated first. This was done by running a principal component analysis chromosome-wise for all markers and by counting the number of eigenvalues that explained 99.5% of the variation of the respective marker data and adding up all chromosome-wise values resulting in q_eff_, which can then be used to divide a defined significance threshold *α* = 0.05 resulting in the adjusted global significance threshold. The effective number of markers ranged between q_eff_ = 56 and q_eff_ = 79 and all population-specific estimates of q_eff_ can be found in Table S12.

## 5. Conclusions

In contrast to many published stem rust seedling resistance genes in wheat, we could identify several QTLs that were associated with adult-plant resistance and only a single gene causing resistance in all plant stages. The QTLs did not result in full resistance, but it can be assumed that they will be more durable as no race specificity is known. Two new gene loci associated with LR could be used to increase the number of different leaf rust resistance genes in the rye breeding pool and to allow the stacking of resistance genes to also reach a higher level of durability here. The results of this study will help to breed stem rust and leaf rust resistant hybrid cultivars in winter rye.

## Figures and Tables

**Table 1:** Generation, breeder, origin and genotyping system for six populations (P1-P6) and one testcross population used in this study.

Pop	Generation	Breeder	Susceptible Parent	Source of SR Resistance Donor	Genotyping System
P1	F_2:5_	KWL	19-70P1_KWL_190	KWL/PET	SNP chip
P2	F_2:5_	KWL	19-70P2_KWL_290	KWL/PET	SNP chip
P2TC	P2 × Tester	KWL	19-70P2_KWL_290	KWL/PET	SNP chip ^a^
P3	F_2:5_	KWL	Lo90	VIR819	SNP chip
P4	BC_1_ F_2:3_	UHOH	L301-N	Elbon	SNP chip
P5	BC_1_ F_2:3_	UHOH	L301-N	Gator	SNP chip
P6	F_2:3_	HYBRO	HSR01	HSR02	SNP DArTseq^TM^

KWL/PET = breeding material from the breeding station in Petkus near Baruth/Mark and belonging to the KWS LOCHOW GmbH; HSR01/HSR02 = breeding material belonging to HYBRO Saatzucht GmbH & Co. KG, Schenkenberg, Germany, VIR819 = genetic resources from the N. I. Vavilov All-Russian Institute of Plant Genetic Resources, Saint Petersburg, Russia; ^a^ The marker data of the respective line population (P2) was used.

**Table 2:** Basic statistics for the different populations (columns) and stem rust severity.

Statistic	P1	P2	P2TC	P3	P4	P5	P6
N_Env_	4	4	3	4	3	3	2
Min	9.0	10.8	20.1	1.6	9.1	3.4	4.5
Max	41.0	58.5	54.2	42.5	46.7	62.6	44.7
Mean	26.7	31.3	38.2	11.4	24.8	25.4	16.0
avSED	5.1	8.8	7.1	4.5	10.2	16.0	10.1
LSD_5%_	10.1	17.3	13.9	8.8	20.1	31.5	19.9

N_Env_ = Number of environments, avSED = average of pairwise standard errors of a difference, LSD_5%_ = least significant difference on a five percent level.

**Table 3:** Variance components (Est.) with standard errors (SE) and entry-mean heritabilities (H^2^) for stem rust.

	P1		P2		P2TC		P3		P4		P5		P6	
	Est.	SE	Est.	SE	Est.	SE	Est.	SE	Est.	SE	Est.	SE	Est.	SE
Genotype (G)	27.9	6.1	60.9	15.1	30.0	8.6	40.4	7.5	22.4	9.1	193.1	49.2	18.5	4.0
Environment (E)	65.8	54.6	402.8	332.5	797.1	799.8	17.2	15.9	423.6	425.3	239.9	244.8	51.3	74.7
Replicate (R)	0.3	4.9	7.4	16.9	2.0	10.7	3.0	7.0	0.0	-	2.7	12.5	0.5	1.8
Block (B)	16.7	9.4	10.3	11.2	13.9	7.2	4.7	3.6	4.8	9.2	0.3	2.0	14.1	9.7
G × E	25.5	4.5	108.6	13.5	48.8	8.0	20.3	3.5	68.8	12.1	353.6	39.7	0.0	-
Residual	55.2	8.1	87.3	14.0	47.6	7.9	33.0	5.2	105.0	16.7	49.7	7.5	127.1	15.1
H^2^	0.68		0.61		0.54		0.80		0.30		0.60		0.27	

Heterogenous variance (population-environment wise) was fitted for the replicate and block effect and the residual and simple means across the levels are reported. No variance components for P4 and P5 and the trait LR are reported because those populations were not segregating in the single locations (low repeatability).

**Table 4:** QTL/Gene effects for stem rust that was artificially inoculated and assessed in the field and associated with respective markers in the different populations.

Pop.	QTL/Gene	Chr.	Marker	Pos. [cM]	*p*-Value a	*p*-Value d	*p*-Value a M × E	*p*-Value d M × E	Het. Effect	SE Het	Hom. Effect	SE Hom	pG
P1	QTL-SR4	3R	isotig16442	78.9	3.5 × 10^−5^	1.2 × 10^−1^	2.5 × 10^−1^	1.3 × 10^−1^	−5.5	4.2	−5.4	4.0	0.24
	QTL-SR5	7R	isotig25901	53.9 *	3.0 × 10^−2^	5.7 × 10^−2^	6.7 × 10^−3^	6.8 × 10^−1^	−1.1	4.2	4.2	4.1	0.09
	QTL-SR4 + QTL-SR5												0.39
P2	QTL-SR6	1R	C9312_837	152.3 *	1.0 × 10^−3^	7.4 × 10^−4^	3.9 × 10^−1^	4.5 × 10^−4^	−4.9	10.6	7.2	10.6	0.28
	QTL-SR4.1	3R	isotig24114	57.5 *	1.4 × 10^−9^	6.2 × 10^−1^	5.0 × 10^−8^	4.0 × 10^−1^	−8.1	12.5	−13.6	12.5	0.4
	QTL-SR4	3R	isotig21556	77.7 *	1.7 × 10^−7^	1.2 × 10^−2^	1.2 × 10^−5^	4.9 × 10^−1^	−10.6	12.5	−11.5	12.5	0.38
	QTL-SR4.1 + QTL-SR4 + QTL-SR6												0.63
P2TC	QTL-SR4.2	3R	C1765_676	120.3 *	4.9 × 10^−4^	9.7 × 10^−3^	5.2 × 10^−1^	8.5 × 10^−2^	−7.2	17.2	−5.8	17.2	0.28
	QTL-SR7	4R	isotig18345	118.0	8.3 × 10^−5^	6.2 × 10^−2^	2.9 × 10^−3^	1.9 × 10^−1^	0.2	17.4	−6.6	17.3	0.26
	QTL-SR8	6R	C3139_486	147.7	1.8 × 10^−3^	2.6 × 10^−2^	1.6 × 10^−2^	1.1 × 10^−3^	−0.2	16.1	5.7	16.1	0.17
	QTL-SR4.2 + QTL-SR7 + QTL-SR8												0.55
P4	QTL-SR9	1R	isotig12035	80.7 *	1.1 × 10^−4^	5.0 × 10^−1^	6.2 × 10^−5^	1.3 × 10^−1^	−6.7	14.3	−12.7	14.4	0.19
	QTL-SR4	3R	isotig19296	85.7 *	1.5 × 10^−7^	3.8 × 10^−3^	1.4 × 10^−6^	1.4 × 10^−1^	−10.9	15.7	−12.1	15.8	0.53
	QTL-SR10	6R	isotig16906	156.9 *	5.0 × 10^−5^	3.5 × 10^−1^	1.0 × 10^−4^	2.4 × 10^−2^	−7.6	15.9	−10.8	15.9	0.25
	QTL-SR4 + QTL-SR9 + QTL-SR10												0.81
P5	*Pgs3.1*	2R	isotig20303	175.2 *	6.9 × 10^−56^	6.6 × 10^−3^	1.1 × 10^−81^	6.5 × 10^−4^	−25.5	20.5	−40.1	20.5	0.82
P6	QTL-SR11	1R	X3575928.F.0.7	67.5 *	5.7 × 10^−9^	1.8 × 10^−1^	6.7 × 10^−16^	1.2 × 10^−2^	−12.1	12.0	−17.4	12.0	0.30
	QTL-SR12	3R	X5802439.F.0.18	48.8 *	1.6 × 10^−4^	8.1 × 10^−2^	4.6 × 10^−2^	5.2 × 10^−1^	−6.1	7.1	−10.0	7.2	0.21
	QTL-SR13	4R	X3357917.F.0.22	70.5	6.2 × 10^−5^	1.5 × 10^−2^	1.4 × 10^−1^	9.5 × 10^−1^	−7.3	7.2	−9.6	7.3	0.26
	QTL-SR14	5R	X3364753.F.0.45	59.8	4.4 × 10^−6^	5.6 × 10^−2^	2.0 × 10^−2^	8.9 × 10^−1^	−8.2	8.4	−12.1	8.4	0.28
	QTL-SR11 + QTL-SR12 + QTL-SR13 + QTL-SR14												0.48

Markers were refenced to chromosomes (Chr.) and positions (Pos.) of the linkage map from Bauer et al. [27]; if not possible (*) the position of another significant marker close by [cM] was reported. *p*-values were extracted from Wald test statistics based on a mixed linear model sequentially fitting effects for an additive (a, A, H, B = 0, 1, 2) and dominant (d, A, H, B = 0, 1, 0) main marker effects followed by respective marker-environment interactions (M × E). Effects and standard errors (SE) for the heterozygous (Het., M_a_ = 1, M_d_ = 1) and the homozygous (Hom., M_a_ = 2, M_d_ = 0) marker state were calculated (predicted) from the mixed linear model. The explained genetic variance (pG) was estimated from the difference of genetic variance in a Null-model without marker effects and a model with the respective marker(s) divided by the genetic variance in the Null-model.

**Table 5:** Results of parental lines tested in the seedling stage with different stem rust isolates (columns).

Pop	Parent	3c-3	3h-3	43-1	6-1	11-4	173-1	174-1
P1	susceptible	5.3	5.0	4.2	4.7	5.0		
	resistant	5.0	5.0	4.7	4.5	4.8		
P2	susceptible	5.0	5.0	4.8	5.0	4.2		
	resistant	5.0	5.0	4.7	4.5	4.8		
P3	susceptible	5.3	5.0	5.0	5.0	5.0	5.0	5.0
	resistant	3.6	4.3	3.8	3.5	4.0	3.0	3.0
P4	susceptible	5.8	5.0	5.0	4.3	5.0	6.0	5.0
	resistant *	4.8	5.0	4.8	5.0	4.2	5.0	5.0
P5	susceptible	5.8	5.0	5.0	4.3	5.0		
	resistant *	1.2	1.8	2.8	2.5	2.5		
P6	susceptible	5.0	5.0	5.0	4.7	5.0	5.0	5.0
	resistant	6.0	4.5	5.0	5.0	5.0	4.0	4.0

The plants were scored according to a 1–6 scale and cell colours indicate resistance (green) and susceptible (red) reaction. For each value, four plants were assessed and the (simple) mean of all scores is reported. * The parental genotype was not available anymore and thus from each population the most resistant genotype across all environments in 2019 (P4_5, P5_9) was reported instead. If cells are empty, the respective genotype-isolate interaction was not tested. Values in bold and with underscore indicate combinations where all genotypes of the respective population were tested in the seedling stage with the respective isolate. In all other cases only the parents were tested.

**Table 6:** Variance components and intercept estimates (Est.) with standard errors (SE) for populations P3, P5, P6 analysed for stem rust with seedling test and estimated by means a cumulative mixed logit model.

Effect		P5		P3		P6	
		Est.	SE	Est.	SE	Est.	SE
Isolate (I)		0.01	0.12	1.89	1.37	n.a.	n.a.
Genotype (G)		6.79	2.61	7.69	2.77	32.01	5.66
G×I		0.71	2.61	n.a.	n.a.	n.a.	n.a.
Tray		0.05	0.23	0.52	0.72	1.41	1.19
Tray position		0.00	0.00	1.24	1.11	0.00	0.00
Intercept	1|2	−8.71	0.71	−9.28	0.89	-	-
	2|3	−4.67	0.34	−6.35	0.78	-	-
	3|4	−1.39	0.31	−1.04	0.73	-	-
	4|5	0.70	0.31	0.47	0.73	−9.74	2.08
	5|6	5.84	0.39	6.21	0.77	-	-

n.a. = no model convergence when this effect was fitted = respective infection types were not scored.

**Table 7:** Basic statistics for the different populations (columns) and leaf rust severity.

Statistic	P1	P2	P2TC	P3	P6
N_Env_	4	6	6	5	1
Min	12.6	5.6	9.9	7.7	−2.9
Max	46.0	38.7	30.2	26.0	65.9
Mean	26.9	17.1	18.7	16.2	14.4
avSED	6.4	5.9	3.2	3.2	6.6
LSD_5%_	12.7	11.6	6.3	6.3	13.2

N_Env_ = Number of environments, avSED = average of pairwise standard errors of a difference, LSD_5%_ = least significant difference on a five percent level.

**Table 8:** Variance components (Est.) with standard errors (SE) and heritability (H^2^) for leaf rust severity.

	P1		P2		P2TC		P3		P6	
	Est.	SE	Est.	SE	Est.	SE	Est.	SE	Est.	SE
Genotype (G)	31.2	7.9	23.8	4.5	25.0	4.5	8.6	1.9	207.5	34.4
Environment (E)	97.9	85.2	60.7	39.9	185.9	119.0	72.8	55.8		
Replicate (R)	6.8	25.9	3.1	12.0	0.4	2.0	12.4	31.1	0.0	-
Block (B)	56.7	21.2	15.1	19.3	9.8	9.2	3.6	6.5	13.6	6.8
G × E	45.4	7.1	10.5	2.2	15.0	1.8	7.3	1.3		
Residual	92.1	13.4	209.3	25.3	132.3	16.6	120.5	14.5	38.4	6.6
H^2^	0.60		0.58		0.83		0.62		0.90	

Heterogenous variance (population-environment wise) was fitted for the replicate and block effect and the residual and simple means across the levels are reported. No variance components for P4 and P5 and the trait LR are reported because those populations were not segregating in the single locations (low repeatability).

**Table 9:** QTL/Gene effects for the naturally occurring leaf rust associated with respective markers in the different populations.

Pop.	QTL/Gene	Chr	Marker	Pos. [cM]	*p*-Value a	*p*-Value d	*p*-Value a M × E	*p*-Value d M × E	Het. Effect	SE Het	Hom. Effect	SE Hom	pG
P1	QTL-LR3	2R	C6172_272	132.4 *	2.2 × 10^−4^	1.4 × 10^−1^	1.7 × 10^−2^	1.1 × 10^−1^	−0.3	5.2	8.6	5.4	0.28
P2	*Pr7*	3R	isotig04119	166.7	7.5 × 10^−22^	2.3 × 10^−2^	9.9 × 10^−36^	8.9 × 10^−4^	−12.7	6.2	−20.7	6.2	0.62
P2TC	*Pr7*	3R	isotig04119	166.7	4.8 × 10^−26^	3.2 × 10^−1^	5.5 × 10^−29^	8.9 × 10^−3^	−6.2	8.2	−17.6	8.2	0.65
P6	*Pr8*	1R	X3358988.F.0.22	139.8 *	3.8 × 10^−29^	7.4 × 10^−13^	n.a.	n.a.	−30.6	3.0	−33.7	2.6	0.74

Markers were refenced to chromosomes (Chr.) and positions (Pos.) of the linkage map from Bauer et al. [27]; if not possible (*) the position of another significant marker close by [cM] was reported. *p*-values were extracted from Wald test statistics based on a mixed linear model sequentially fitting effects for an additive (a; A, H, B = 0, 1, 2) and dominant (d; A, H, B = 0, 1, 0) main marker effects followed by respective marker-environment interactions (M × E). Effects and standard errors (SE) for the heterozygous (Het., M_a_ = 1, M_d_ = 1) and the homozygous (Hom., M_a_ = 2, M_d_ = 0) marker state were calculated (predicted) from the mixed linear model. The explained genetic variance (pG) was estimated from the difference of genetic variance in a Null-model without marker effects and a model with the respective marker(s) divided by the genetic variance in the Null-model. P6 was tested in only one environment and thus M × E could not be estimated (n.a.).

## Data Availability

Genotypes and marker data are proprietary materials of KWS LOCHOW GmbH (P1-P3, P2TC) and HYBRO Saatzucht GmbH & Co. KG (P6). Segregating plant material is available on request to these companies for scientists without any commercial interest. A respective MTA must be signed in advance. Breeding material developed at the University of Hohenheim (population P4, P5) is freely available from the corresponding author.

## Supplementary Materials

The supporting information can be downloaded at: https://www.mdpi.com/article/10.3390/ijms232213674/s1.

## Abbreviations

APR	adult-plant resistance
ASR	all-stage resistance
BLUEs	best linear unbiased estimators
BLUPs	best linear unbiased predictors
CMS	cytoplasmatic-male sterility
LR	leaf rust
LS	leaf spots
SR	stem rust
ST	seedling test
