# Supplementary material for: Studying Stem Rust and Leaf Rust Resistances of Self-Fertile Rye Breeding Populations
Source: Int J Mol Sci. 2022 Nov 8;23(22):13674. doi: 10.3390/ijms232213674 (PMC9692268; doi:10.3390/ijms232213674)
Supplement: Supplementary file 1 [file ijms-23-13674-s001.zip › ijms-1963772-supplementary.pdf]

Supplement

Studying stem rust and leaf rust resistances of self-fertile rye breeding populations

Paul Gruner[1], Anne Witzke[2], Kerstin Flath[2], Jakob Eifler[3], Brigitta Schmiedchen[3], Malthe Schmidt[3], Andres Gordillo[3], Dörthe Siekmann[4], Franz Joachim Fromme[4], Silvia Koch[1], Hans Peter Piepho[5], Thomas Miedaner[1*]

[1] University of Hohenheim, State Plant Breeding Institute, 70593 Stuttgart, Germany

[2] Julius Kuehn-Institute, Institute for Plant Protection in Field Crops and Grassland, Stahnsdorfer Damm 81, 14532 Kleinmachnow, Germany

[3] KWS LOCHOW GmbH, Ferdinand-von-Lochow-Str. 5, 29296 Bergen, Germany

[4] HYBRO Saatzucht GmbH & Co. KG, Kleptow 53, 17291 Schenkenberg

[5] University of Hohenheim, Biostatistics Unit, Institute of Crop Science, 70593 Stuttgart, Germany

*Corresponding author: Thomas Miedaner; miedaner@uni-hohenheim.de; +49 711 45922690

Supplementary Tables

Repeatabilitiy stem rust

Table S1 Environment- and population-wise repeatabilties of the best stem rust (%) assessments.

Set	HOH		DAH		PET	WOH	KLE	
	**2019**	**2020**	**2019**	**2020**	**2019**	**2019**	**2019**	**2020**
	SR3	SR2	SR3	SR3	SR2	SR3	SR3	SR3
**P1**			0.71	0.80	0.63	0.59		
**P2**	0.95	0.74			0.74	0.44		
**P2TC**	0.91				0.74	0.45		
**P3**			0.89	0.83	0.55	0.82		
**P4**	0.88	0.82					0.60	
**P5**	0.98	0.91					0.83	
**P6**		0.84						0.58

HOH = Stuttgart-Hohenheim, EWE = Eckartsweier near Willstätt, DAH = Berlin-Dahlem, PET = Petkus near Baruth/Mark, WOH = Wohlde near Bergen, KLE = Kleptow near Prenzlau, WUL = Wulfsode near Wriedel

Variance components bivariate model

Table S2 Variance components (Est.) with standard errors (SE) for a bivariate mixed model fit of a line population (P2) and the respective testcrosses (P2TC) for the traits stem rust and leaf rust.

**Component**	**Stem rust**		**Leaf rust**	
	**Est.**	**SE**	**Est.**	**SE**
Environment (E) P2	404.1	333.4	55.1	36.4
E P2TC	796.8	799.6	185.7	118.8
Genotype (G) P2	62.6	15.5	24.5	4.6
G P2TC	30.2	8.5	27.1	4.9
Covariance (Correlation) G P2-P2TC	28.6 (0.66)	8.9 (0.14)	24.9 (0.97)	4.4 (0.03)
GxE P2	109.8	13.6	11.0	2.1
GxE P2TC	47.7	7.9	15.6	1.9
Covariance (Correlation) GxE P2-P2TC	20.8 (0.29)	7.4 (0.10)	8.8 (0.67)	1.6 (0.08)
Replicate P2	6.2	11.0	2.7	4.2
Replicate P2TC	6.2	11.0	2.7	4.2
Block P2	44.5	22.3	58.5	53.3
Block P2TC	44.5	22.3	58.5	53.3
Residual P2	143.7	23.6	760.1	95.7
Residual P2TC	143.7	23.6	760.1	95.7

Unstructured (general correlation) variance-covariance was fitted for the genotype and the genotype-environment interaction. Separate variances were fitted for the environment effect of lines and testcrosses. Heterogenous variance on an environment- and set-wise level was allowed for the replicate and block effects as well as for the residual. Simple means across levels and for the respective sets are reported.

Marker effects SR

Table S3 QTL/Gene effects for the trait stem rust associated with respective markers in the different populations.

Pop.	QTL/ Gene	Chr.	Marker	Pos. [cM]	CI left LOD-1	CI right LOD-1	P-value a	P-value d	P-value a M×E	P-value d M×E	Het. effect	SE Het	Hom. effect	SE Hom	N(allele)			pG
															A	H	B	
P1	QTL-SR4	3R	isotig16442	78.9	77.7	83.3	3.5E-05	1.2E-01	2.5E-01	1.3E-01	-5.5	4.2	-5.4	4.0	32	14	25	0.24
P1	QTL-SR4	3R	isotig33162	86.5	86.1	89.3	4.0E-04	5.2E-01	7.3E-01	7.2E-02	-4.2	4.3	-4.9	4.1	36	15	18	0.16
P1	QTL-SR5	7R	isotig25901	53.9*	52.3	54.3	3.0E-02	5.7E-02	6.7E-03	6.8E-01	-1.1	4.2	4.2	4.1	26	17	30	0.09
P1	QTL-SR4 + QTL-SR5																	0.39
P2	QTL-SR6	1R	C9312_837	152.3*	145.9	153.5	1.0E-03	7.4E-04	3.9E-01	4.5E-04	-4.9	10.6	7.2	10.6	28	20	36	0.28
P2	QTL-SR4.1	3R	isotig24114	57.5*	20.7	65.9	1.4E-09	6.2E-01	5.0E-08	4.0E-01	-8.1	12.5	-13.6	12.5	23	28	32	0.40
P2	QTL-SR4	3R	isotig21556	77.7*	77.3	78.9	1.7E-07	1.2E-02	1.2E-05	4.9E-01	-10.6	12.5	-11.5	12.5	28	26	31	0.38
P2	QTL-SR4	3R	isotig33162	86.5	74.5	92.2	2.3E-05	2.0E-01	4.0E-04	9.3E-01	-7.7	12.2	-10.3	12.2	25	29	32	0.24
P3	QTL-SR4.1 + QTL-SR4 + QTL-SR6																	0.63
P2TC	QTL-SR4.2	3R	C1765_676	120.3*	118.7	137.7	4.9E-04	9.7E-03	5.2E-01	8.5E-02	-7.2	17.2	-5.8	17.2	33	19	34	0.28
P2TC	QTL-SR4	3R	isotig33162	86.5	0.0	101.3	8.0E-03	6.8E-01	2.6E-02	1.7E-01	-1.5	17.1	-4.4	17.1	25	29	32	0.07
P2TC	QTL-SR7	4R	isotig18345	118.0	115.2	130.8	8.3E-05	6.2E-02	2.9E-03	1.9E-01	0.2	17.4	-6.6	17.3	33	16	33	0.26
P2TC	QTL-SR8	6R	C3139_486	147.7	138.2	156.5	1.8E-03	2.6E-02	1.6E-02	1.1E-03	-0.2	16.1	5.7	16.1	24	23	35	0.17
	QTL-SR4.2 + QTL-SR7 + QTL-SR8																	0.55
P4	QTL-SR9	1R	isotig12035	80.7*	78.3	84.8	1.1E-04	5.0E-01	6.2E-05	1.3E-01	-6.7	14.3	-12.7	14.4	14	58	15	0.19
P4	QTL-SR4	3R	isotig19296	85.7*	76.1	86.1	1.5E-07	3.8E-03	1.4E-06	1.4E-01	-10.9	15.7	-12.1	15.8	23	41	22	0.53

Pop.	QTL/ Gene	Chr.	Marker	Pos. [cM]	CI left LOD-1	CI right LOD-1	P-value a	P-value d	P-value a M×E	P-value d M×E	Het. effect	SE Het	Hom. effect	SE Hom	N(allele)			pG
P4	QTL-SR4	3R	isotig33162	86.5	7.2	88.9	4.0E-04	3.6E-02	2.2E-08	4.2E-02	-9.1	16.3	-9.8	16.3	20	41	24	0.14
P4	QTL-SR10	6R	isotig16906	156.9*	154.9	162.1	5.0E-05	3.5E-01	1.0E-04	2.4E-02	-7.6	15.9	-10.8	15.9	15	41	30	0.25
P4	QTL-SR4 + QTL-SR9																	0.76
P4	QTL-SR4 + QTL-SR9 + QTL-SR10																	0.81
P5	Pgs3.1	2R	isotig20303	175.2*	165.8	175.2	6.9E-56	6.6E-03	1.1E-81	6.5E-04	-25.5	20.5	-40.1	20.5	26	39	20	0.82
P6	QTL-SR11	1R	X3575928.F.0.7	67.5*	66.3	67.9	5.7E-09	1.8E-01	6.7E-16	1.2E-02	-12.1	12.0	-17.4	12.0	25	48	18	0.30
P6	QTL-SR12	3R	X5802439.F.0.18	48.8*	47.6	49.6	1.6E-04	8.1E-02	4.6E-02	5.2E-01	-6.1	7.1	-10.0	7.2	12	56	22	0.21
P6	QTL-SR13	4R	X3357917.F.0.22	70.5	68.2	71.8	6.2E-05	1.5E-02	1.4E-01	9.5E-01	-7.3	7.2	-9.6	7.3	11	56	21	0.26
P6	QTL-SR14	5R	X3364753.F.0.45	59.8	59.4	60.2	4.4E-06	5.6E-02	2.0E-02	8.9E-01	-8.2	8.4	-12.1	8.4	11	52	28	0.28
P6	QTL-SR11 + QTL-SR12 + QTL-SR13 + QTL-SR14																	0.48

Markers were referenced to chromosomes (Chr.) and positions (Pos.) of the linkage map from Bauer et al. (2017); if not possible (*) the position of another significant marker close by [cM] was reported. Confidence intervals (CI LOD-1) were determined by choosing the position of the first marker to the left or the right on the consensus map for which the LOD ((-$\log_{10}$(p-value)) dropped by at least one. P-values were extracted from Wald test statistics based on a mixed linear model sequentially fitting effects for an additive (a, A,H,B = 0,1,2) and dominant (d, A,H,B = 0,1,0) main marker effect followed by respective marker-environment interactions (M×E). Effects and standard errors (SE) for the heterozygous (Het., M_a = 1, M_d = 1) and the homozygous (Hom., M_a = 2, M_d = 0) marker state were calculated (predicted) from the mixed linear model. The explained genetic variance (pG) was estimated by the difference of genetic variance in a Null-model without marker effects and the model with the respective marker(s) divided by the genetic variance in the Null-model. The number of genotypes with the marker alleles (A,H,B) were listed (N(allele))

Common marker QTL-SR4

Table S4 Common marker for QTL-SR4: isotig33162 (3R, 86.5cM).

Pop	P-value a	P-value d	P-value a M×E	P-value d M×E	Het. effect	SE Het	Hom. effect	SE Hom	N(allele)			pG
									A	H	B	
**P1**	4.0E-04	5.2E-01	7.3E-01	7.2E-02	-4.2	4.3	-4.9	4.1	36	15	18	0.16
**P2**	2.3E-05	2.0E-01	4.0E-04	9.3E-01	-7.7	12.2	-10.3	12.2	25	29	32	0.24
**P2TC**	8.0E-03	6.8E-01	2.6E-02	1.7E-01	-1.5	17.1	-4.4	17.1	25	29	32	0.07
**P4**	4.0E-04	3.6E-02	2.2E-08	4.2E-02	-9.1	16.3	-9.8	16.3	20	41	24	0.14

P-values were extracted from Wald test statistics based on a mixed linear model sequentially fitting effects for an additive (a, A,H,B = 0,1,2) and dominant (d, A,H,B = 0,1,0) main marker effect followed by respective marker-environment interactions (M×E). Effects and standard errors (SE) for the heterozygous (Het., $M_a = 1$, $M_d = 1$) and the homozygous (Hom., $M_a = 2$, $M_d = 0$) marker state were calculated (predicted) from the mixed linear model. The explained genetic variance (pG) was estimated by the difference of genetic variance in a Null-model without marker effects and the model with the respective marker(s) divided by the genetic variance in the Null-model. The number of genotypes with the marker alleles (A,H,B) were listed (N(allele)).

Repeatability leaf rust

Table S5 Environment- and population-wise repeatabilties of the best leaf rust (%) assessments.

Set	HOH		DAH	PET		WOH	
	**2019**	**2020**	**2020**	**2019**	**2020**	**2019**	**2020**
**P1**				0.86	0.88	0.29	0.63
**P2**	0.81	0.83		0.79	0.72	0.76	0.86
**P2TC**	0.75	0.85		0.86	0.94	0.65	0.86
**P3**			0.53	0.79	0.83	0.72	0.86
**P6**		0.90					

HOH = Stuttgart-Hohenheim, EWE = Eckartsweier near Willstätt, DAH = Berlin-Dahlem, PET = Petkus near Baruth/Mark, WOH = Wohlde near Bergen, KLE = Kleptow near Prenzlau, WUL = Wulfsode near Wriedel

Marker effects LR

Table S6 QTL/Gene effects for the trait leaf rust associated with respective markers in the different populations.

Pop.	QTL/ Gene	Chr.	Marker	Pos. [cM]	CI left LOD-1	CI right LOD-1	P-value a	P-value d	P-value a M×E	P-value d M×E	Het. effect	SE Het	Hom. effect	SE Hom	N(allele)			pG
															A	H	B	
P1	QTL-LR3	2	C6172_272	132.4*	131.2	134.4	2.2E-04	1.4E-01	1.7E-02	1.1E-01	-0.3	5.2	8.6	5.4	40	11	21	0.28
P2	*Pr7*	3	isotig04119	166.7	151.8	166.7	7.5E-22	2.3E-02	9.9E-36	8.9E-04	-12.7	6.2	-20.7	6.2	21	33	32	0.62
P2TC	*Pr7*	3	isotig04119	166.7	151.8	166.7	4.8E-26	3.2E-01	5.5E-29	8.9E-03	-6.2	8.2	-17.6	8.2	21	33	32	0.65
P6	*Pr8*	1	X3358988.F.0.22	139.8*	138.2	141.0	3.8E-29	7.4E-13	n.a.	n.a.	-30.6	3.0	-33.7	2.6	17	50	24	0.74

Markers were referenced to chromosomes (Chr.) and positions (Pos.) of the linkage map from Bauer et al. (2017); if not possible (*) the position of another significant marker close by [cM] was reported. Confidence intervals (CI LOD-1) were determined by choosing the position of the first marker to the left or the right on the consensus map for which the LOD ((-$\log_{10}$(p-value)) dropped by at least one. P-values were extracted from Wald test statistics based on a mixed linear model sequentially fitting effects for an additive (a, A,H,B = 0,1,2) and dominant (d, A,H,B = 0,1,0) main marker effect followed by respective marker-environment interactions (M×E). Effects and standard errors (SE) for the heterozygous (Het., M_a = 1, M_d = 1) and the homozygous (Hom., M_a = 2, M_d = 0) marker state were calculated (predicted) from the mixed linear model. The explained genetic variance (pG) was estimated by the difference of genetic variance in a Null-model without marker effects and the model with the respective marker(s) divided by the genetic variance in the Null-model. The number of genotypes with the marker alleles (A,H,B) were listed (N(allele)). The leaf rust resistance was only mapped in a single location and consequently marker-environment interaction could not be estimated (n.a.).

Isolates

Table S7 Virulence reaction of single pustule isolates (rows) on 15 rye differential lines (columns). Those lines have been used (Miedaner et al., 2016) and still are to distinguish stem rust isolates collected in Germany and Poland.

		Differential lines														
		D26	D31	D36	D30	D48	D25	D35	D47	D44	D37	D45	D41	D33	D46	D43
Isolates	3c-3[a]	1	1	1	1	6	1	5	1	3	5	5	5	5	5	5
	3h-3[a]	1	3	1	5	1	4	3	5	5	5	3	3	5	6	6
	43-1[a]	1	1	3	1	1	5	5	1	5	6	5	5	5	5	5
	11-4	1	1	1	1	2	6	4	5	6	4	4	5	5	6	6
	173-1	1	2	1	1	1	3	3	5	4	3	6	6	1	2	6
	174-1	1	1	1	1	2	1	4	1	3	4	3	3	4	4	5

Infection types according to the scale used in this study. [a]Isolates were used for field inoculation.

Seedling test – Sample size

Table S8 Number of plants per genotype used for seedling tests of the respective populations (P3, P5, P6).

Isolate	Parameter	P3	P5	P6
11-4	Min.	1	2	
	Max.	5	6	
	Mean	2.8	4.5	
43-1	Min.	1	1	
	Max.	6	5	
	Mean	2.8	4.6	
3c-3	Min.	1	2	
	Max.	5	6	
	Mean	2.9	4.7	
173-1	Min.	1		4
	Max.	6		8
	Mean	3.5		7.3
174-1	Min.			3
	Max.			8
	Mean			7.4
3h-3	Min.	1	1	
	Max.	5	5	
	Mean	3.1	3.9	

If cells are empty, the respective population-isolate combination was not tested.

Marker and maps

Table S9 Physical position in the Lo7 reference sequence (https://galaxy-web.ipk-gatersleben.de/; Rabanus-Wallace et al. 2021)

Marker	Chr. LM	Chr. Lo7	Pos. Lo7
C1765_676	3R	3R	913,221,061
C26774_197	2R	2R	937,908,614
C3139_486	6R	Un	70,932,245
C31858_239	3R	3R	922,333,044
C6172_272	2R	2R	904,440,000
C9312_837	1R	Un	71,617,927
isotig04119	3R	3R	964,894,000
isotig12035	1R	1R	543,948,000
isotig12934	7R	7R	885,761,000
isotig14534	3R	3R	40,352,000
isotig16442	3R	3R	679,965,000
isotig16906	6R	6R	839,981,000
isotig18345	4R	4R	746,053,000
isotig18857	5R	5R	837,416,000
isotig19296	3R	3R	683,104,000
isotig19591	5R	5R	37,655,000
isotig20203	3R	1R	693,465,000
isotig20303	2R	2R	943,101,000
isotig21556	3R	3R	677,372,000
isotig23465	1R	3R	934,276,000
isotig24114	3R	Un	20,211,000
isotig24825	3R	3R	24,794,000
isotig25901	7R	7R	72,166,000
isotig27039	4R	4R	709,499,000
X3575928.F.0.7	1R	1R	424,464,852
X3357917.F.0.22	4R	4R	394,229,002
X3358988.F.0.22	1R	1R	713,072,950
X3364643.F.0.52	7R	7R	172,737,157
X3364753.F.0.45	5R	5R	484,322,230
X5802439.F.0.18	3R	-	-

Chr. LM = chromosome according to the linkage maps, Chr. Lo7 = chromosome based on BLAST in Lo7 reference sequence, Un = marker sequences BLAST resulted in position on unknown linkage group, Pos. Lo7 = physical position in Lo7 reference sequence ± 1kb, - = marker sequence could not be found in reference sequence

Table S10 Marker statistics

Pop	Marker system	N_{Geno} MAF>0.1 CR>0.9	N_{Marker} CR > 0.95	N_{Marker} redundant	Marker (%)			
					**Allele A**	**Allele B**	**heterozygous**	**missing**
P1	SNP chip	87	1680	655	40.4	30.7	25.9	3.0
P2	SNP chip	87	1519	823	33.7	36.7	26.7	3.0
P3	SNP chip	82	1379	393	40.9	36.5	19.1	3.5
P4	SNP chip	90	1332	995	28.8	19.8	48.7	2.8
P5	SNP chip	87	1198	899	22.3	21.4	53.8	2.5
P6	SNP DArTseq™	91	4002	650	20.8	26.1	52.9	0.2

N_{Geno} = Number of inbred lines after filtering for minor allele frequency (MAF) of 0.1, N_{Marker} = Number of markers with call rate (CR) > 0.95 and the redundant (correlation > 0.99) markers thereof

Table S11 Number of markers (N_M) and maximum length (cM) of linkage maps for all populations (P1-P6)

Chr.	P1		P2		P3		P4		P5		P6		
	N_M	cM	N_M	cM	N_M	cM	N_M	cM	N_M	cM	N_M	cM before*	cM after*
1R	369	318.2	174	199.6	190	223.6	303	122.2	57	57.7	520	1271.6	206.5
2R	365	223.8	478	248.9	251	283	0	0	20	26.3	704	1622.5	197.4
3R	280	307.2	317	240.2	135	187.4	409	150	349	131.9	509	1187.3	147.7
4R	248	272.1	377	224.7	176	223.3	329	163.9	270	127.1	267	776.4	140.2
5R	469	353.3	393	240	273	293.8	260	157.5	331	135.3	900	2381.1	289.6
6R	342	414.9	213	205.3	151	189	360	149.1	327	81.5	614	1673.1	192.7
7R	425	342.4	443	223.5	139	233.4	436	140.1	384	126.6	272	714.2	97.5

*after construction of a first (before) linkage map the genotypic data was “polished” and a second (after) linkage map was constructed

Table S12 Effective marker number estimated for adjustment of the global significance threshold by the simple M method proposed in Gao et al. (2008)

Pop	q_{eff}
P1	60
P2	61
P3	56
P4	59
P5	58
P6	79

Supplementary figures

Manhattan plot SR with cofactors

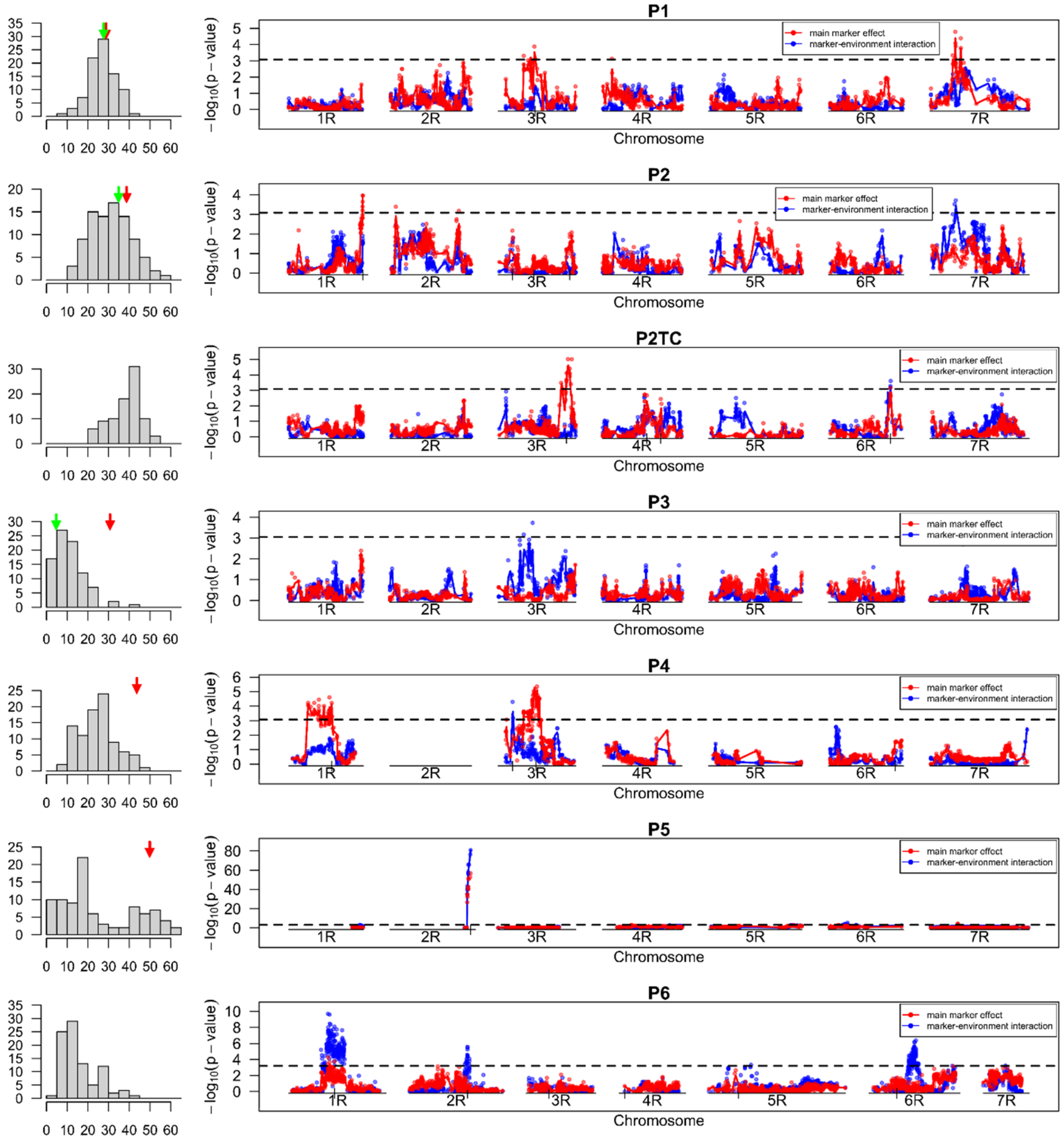

Figure S1 Histograms and LOD (-log$_{10}$(p-value)) profiles for populations P1-P6 and P2TC for stem rust. P-values of the were calculated from a mixed model fitting marker-effect (red) and marker-environment effects (blue) sequentially as fixed effects. Additional markers from a previous marker scan were additionally used as cofactors and the respective positions of those are marked as ticks on the base line. Markers with linkage disequilibrium below 20cM were not used as cofactors for the specific markers displayed here. Marker order of P1-P5 and P2TC was based on a consensus map from linkage maps of all populations (and populations from previous projects). Marker order of P6 was calculated separately as a different marker system was used. The dashed line shows the global significance threshold (alpha = 0.05) adjusted for multiple testing by simpleM method. Histograms are based on best linear estimators (stem rust infection in % on x-axis) of the genotypes used for mapping. The red and green arrows indicate the value of the susceptible and resistant parent respectively; if not presented, it was not tested in the field.

Correlation seedling test vs. field

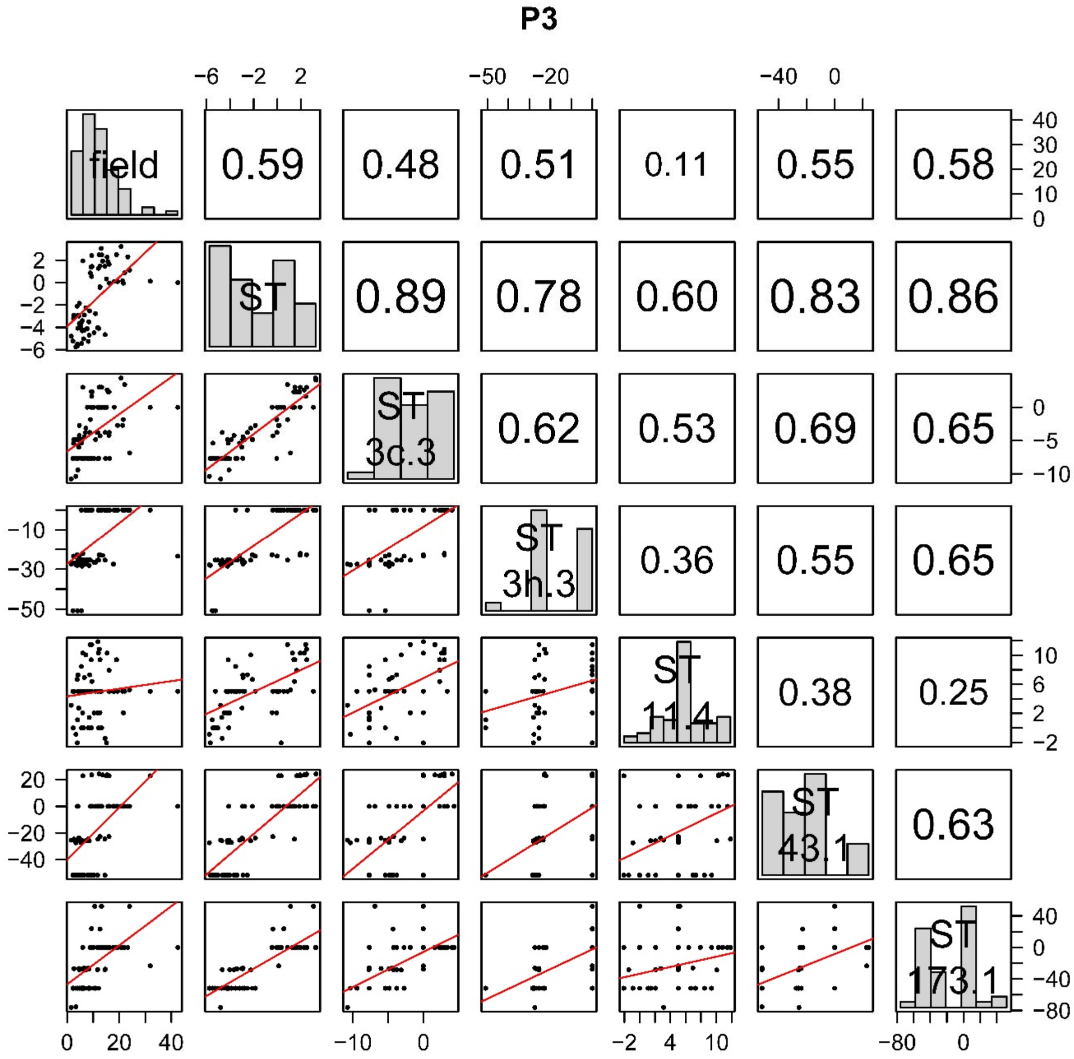

Figure S2 Correlation (plot and Pearson correlation estimate) of best linear unbiased estimators of genotypes from P3 tested in the field (field) and in the seedling stage with different isolates (11.4, 43.1, 3c.3, 3h.3, 173.1) separately. Adjusted means of genotypes for the seedling data were based on with cumulative logit models for isolates separately and combining data from all isolates (ST).

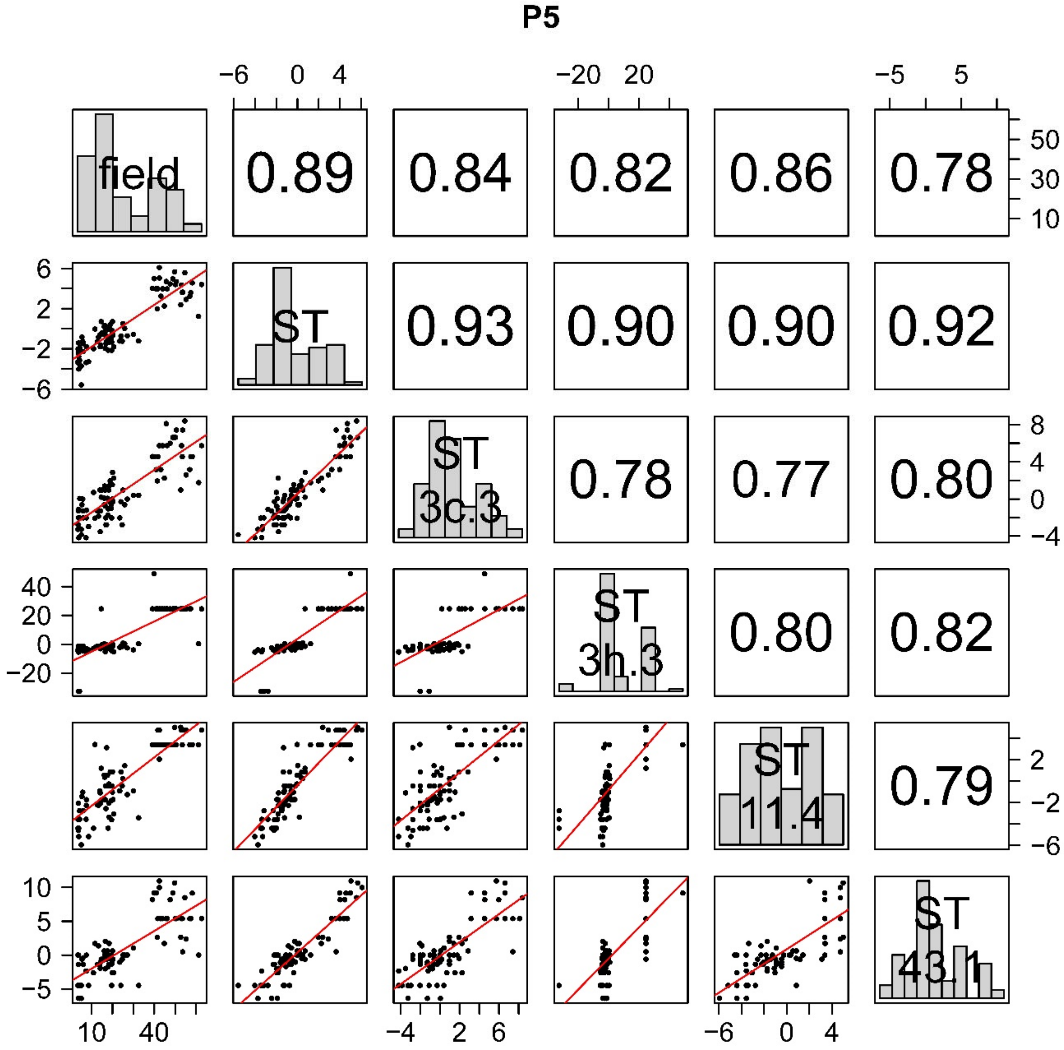

Figure S3 Correlation (plot and Pearson correlation estimate) of best linear unbiased estimators of genotypes from P5 tested in the field (field) and in the seedling stage with different isolates (11.4, 43.1, 3c.3, 3h.3) separately. Adjusted means of genotypes for the seedling data were based on with cumulative logit models for isolates separately and combining data from all isolates (ST).

Manhattanplot isolate-wise seedling test

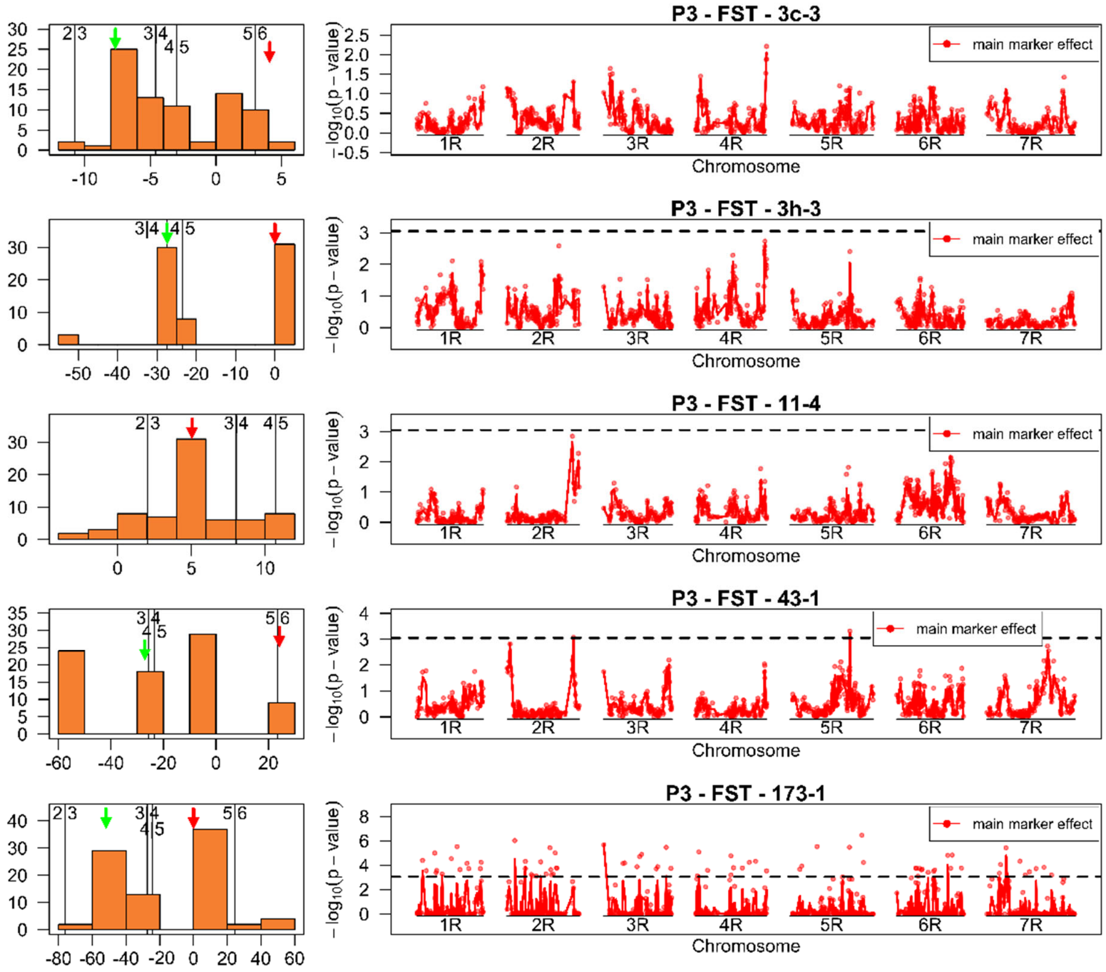

Figure S4 Histograms (left) and LOD-profiles for mapping of seedling resistance in in P3. The histograms are based on the model coefficients of the genotypes fitted in a cumulative logit link model (logit scale). The vertical lines display the intercepts (thresholds) for the respective groups (2|3, 3|4, 4|5, 5|6). Marker-wise p-values are plotted in the LOD profile that were calculated by comparing likelihood differences (ANOVA) of a cumulative logit link model with additive (A,H,B = 0,1,2) and dominant (A,H,B = 0,1,0) marker effects (model b) with a model without marker effects (model a, main effect). Marker order was based on a consensus map from linkage maps of all populations (and populations from previous projects). The dashed line shows the global significance threshold (alpha = 0.05) adjusted for multiple testing by simpleM method.

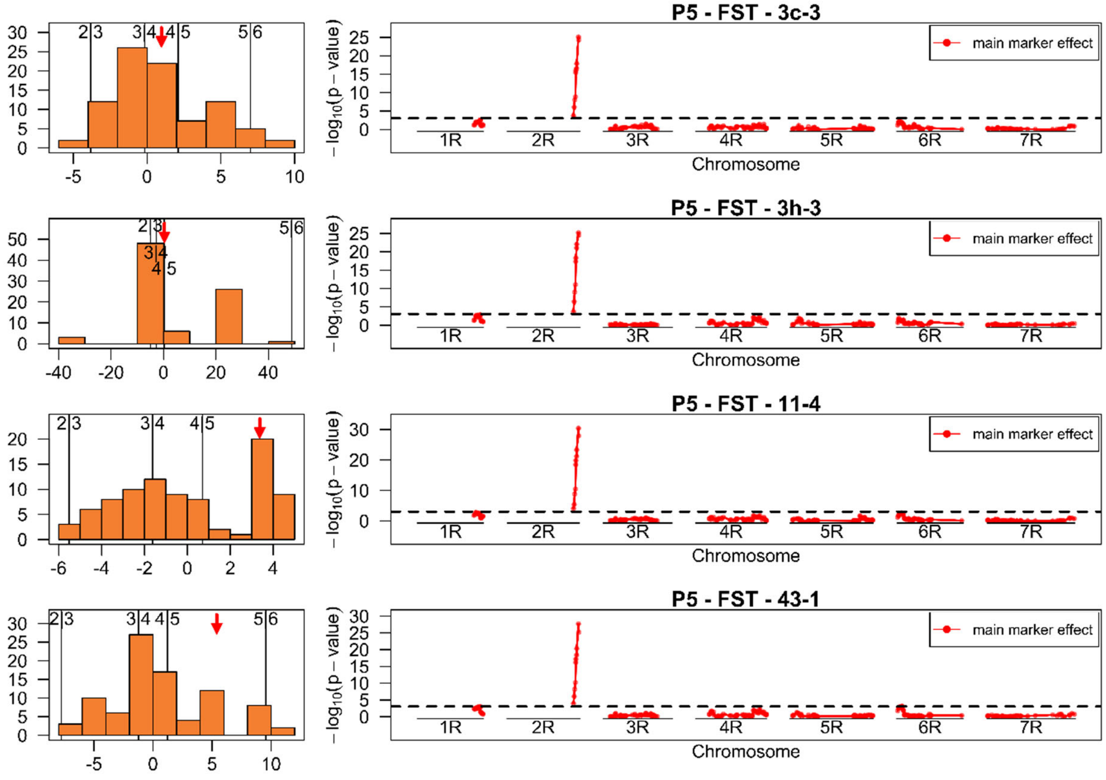

Figure S5 Histograms (left) and LOD-profiles for mapping of seedling resistance in in P5. The histograms are based on the model coefficients of the genotypes fitted in a cumulative logit link model (logit scale). The vertical lines display the intercepts (thresholds) for the respective groups (2|3, 3|4, 4|5, 5|6). Marker-wise p-values are plotted in the LOD profile that were calculated by comparing likelihood differences (ANOVA) of a cumulative logit link model with additive (A,H,B = 0,1,2) and dominant (A,H,B = 0,1,0) marker effects (model b) with a model without marker effects (model a, main effect). Marker order was based on a consensus map from linkage maps of all populations (and populations from previous projects). The dashed line shows the global significance threshold (alpha = 0.05) adjusted for multiple testing by simpleM method.

Manhattan plot LR with cofactors

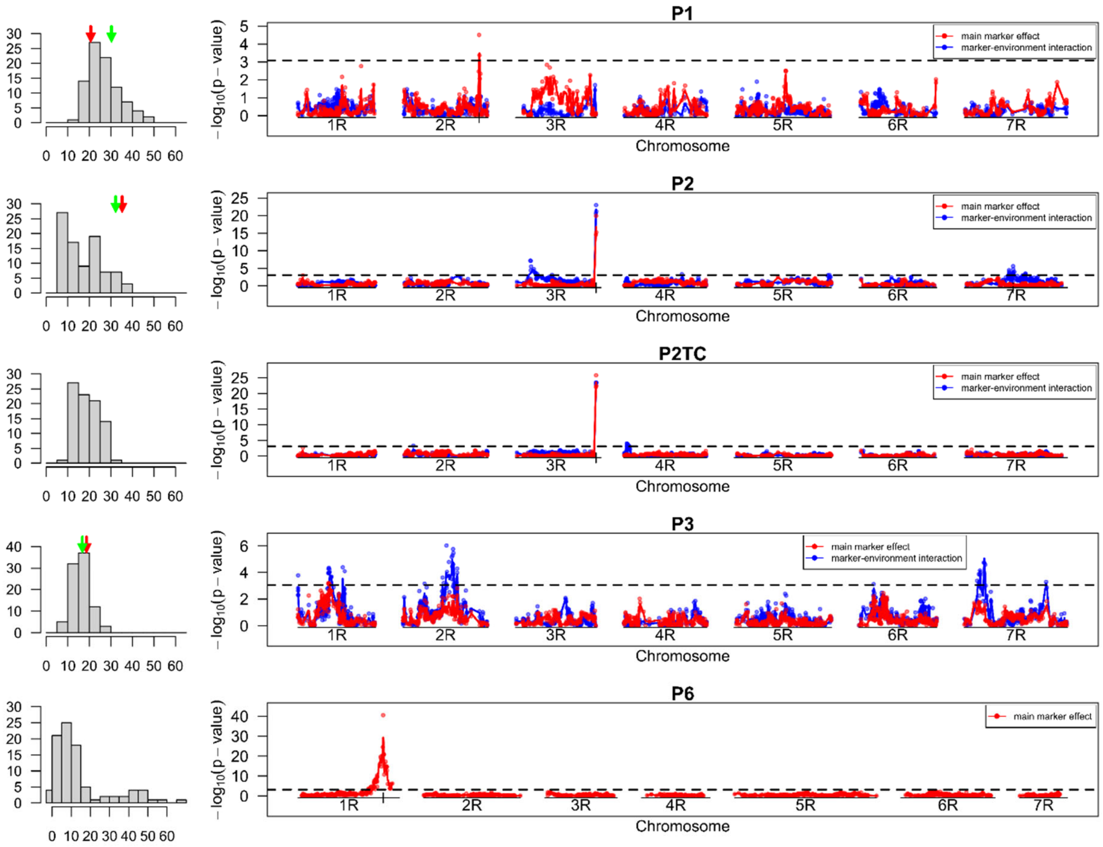

Figure S6 Histograms and LOD (-log$_{10}$(p-value)) profiles for populations P1-P3, P6 and P2TC for leaf rust. P-values of the were calculated from a mixed model fitting marker-effect (red) and marker-environment effects (blue) sequentially as fixed effects. Additional markers from a previous marker scan were additionally used as cofactors and the respective positions of those are marked as ticks on the base line. Markers with linkage disequilibrium below 20cM were not used as cofactors for the specific markers displayed here. Marker order of P1-P5 and P2TC was based on a consensus map from linkage maps of all populations (and populations from previous projects). Marker order of P6 was calculated separately as a different marker system was used. The dashed line shows the global significance threshold (alpha = 0.05) adjusted for multiple testing by simpleM method. Histograms are based on best linear estimators (stem rust infection in % on x-axis) of the genotypes used for mapping. The red and green arrows indicate the value of the (stem rust) susceptible and resistant parent respectively; if not presented, it was not tested in the field.

Linkage map comparison

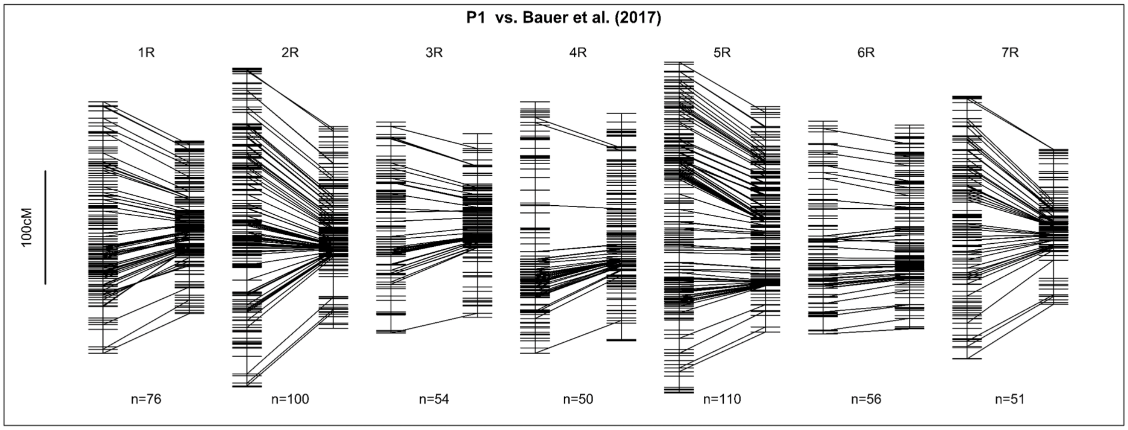

Figure S7 Linkage map comparison (chromosome 1R-7R) of overlapping markers (connected lines) between constructed linkage map based on P1 (left) with the linkage map from Bauer et al. (2017). The number of common markers (n) is reported.

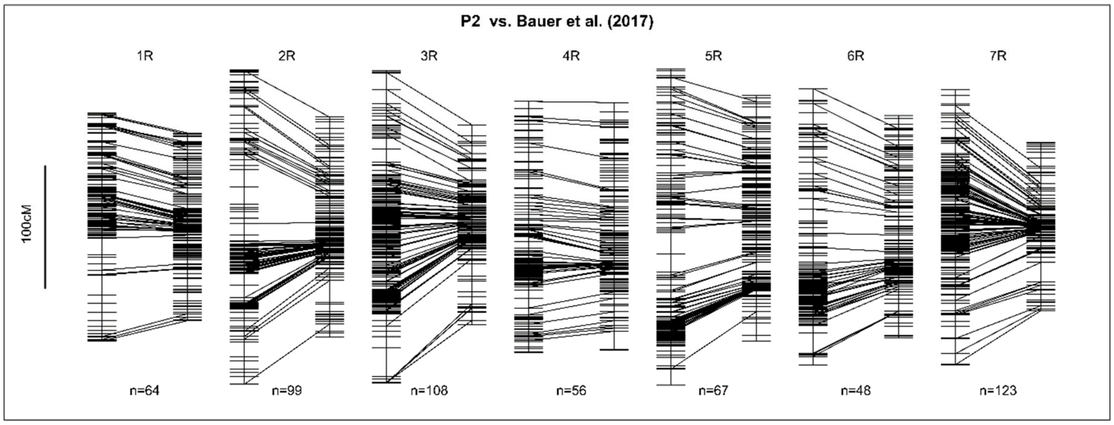

Figure S8 Linkage map comparison (chromosome 1R-7R) of overlapping markers (connected lines) between constructed linkage map based on P2 (left) with the linkage map from Bauer et al. (2017). The number of common markers (n) is reported.

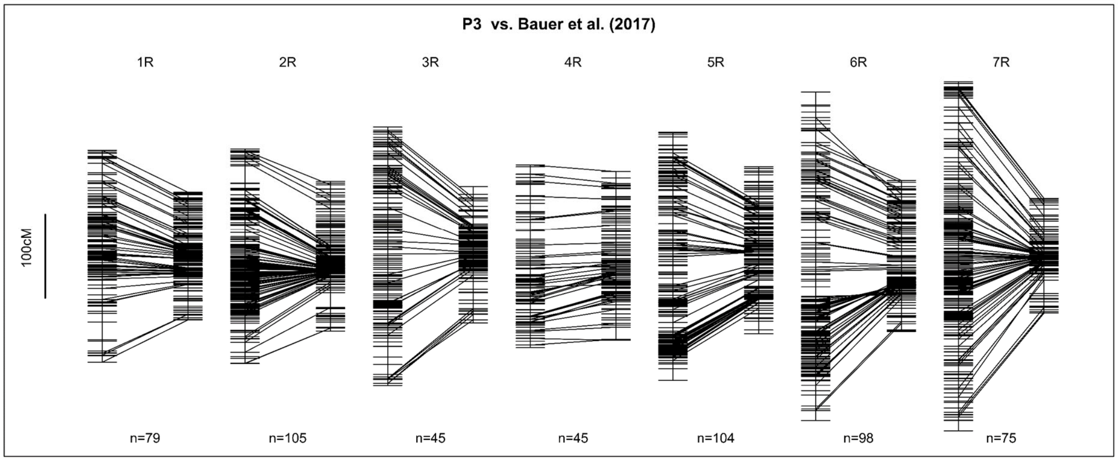

Figure S9 Linkage map comparison (chromosome 1R-7R) of overlapping markers (connected lines) between constructed linkage map based on P3 (left) with the linkage map from Bauer et al. (2017). The number of common markers (n) is reported.

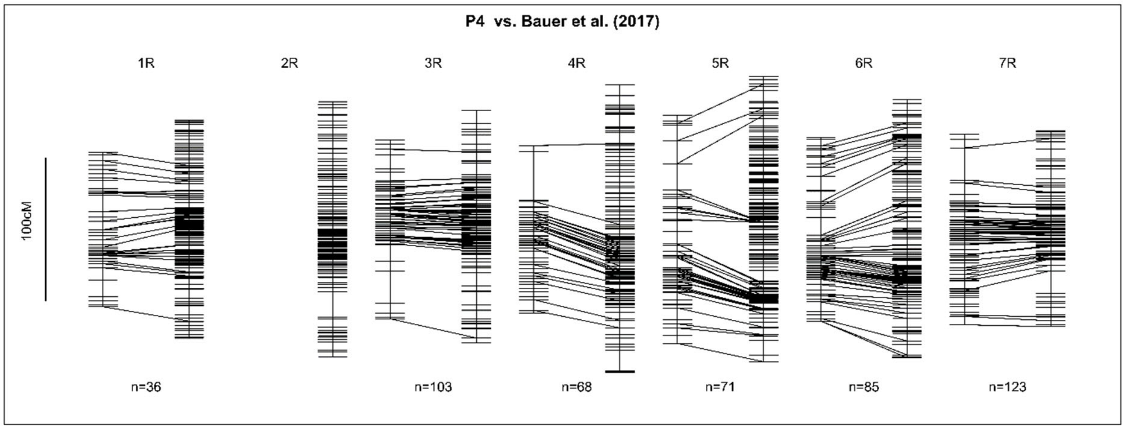

Figure S10 Linkage map comparison (chromosome 1R-7R) of overlapping markers (connected lines) between constructed linkage map based on P4 (left) with the linkage map from Bauer et al. (2017). The number of common markers (n) is reported. P4 was monomorphic for markers of linkage group 2R, thus no map can be displayed for this chromosome.

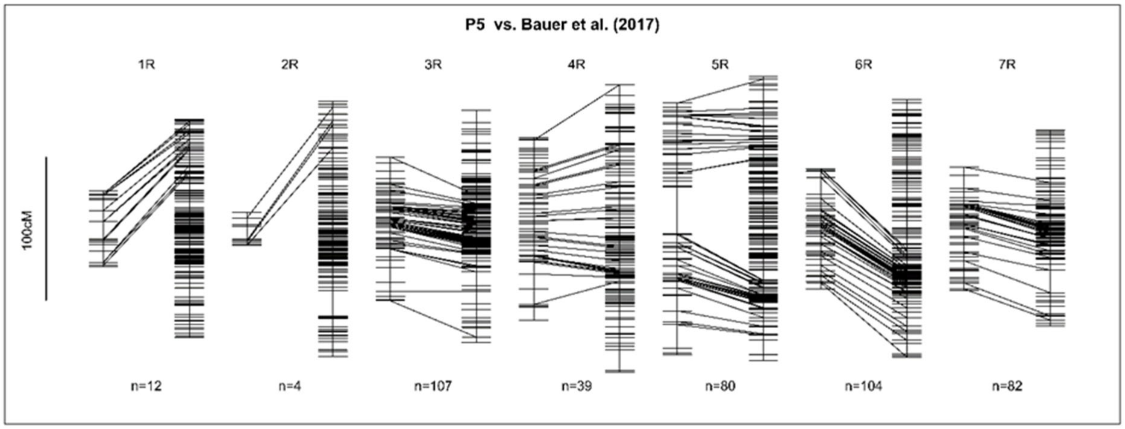

Figure S11 Linkage map comparison (chromosome 1R-7R) of overlapping markers (connected lines) between constructed linkage map based on P5 (left) with the linkage map from Bauer et al. (2017). The number of common markers (n) is reported.

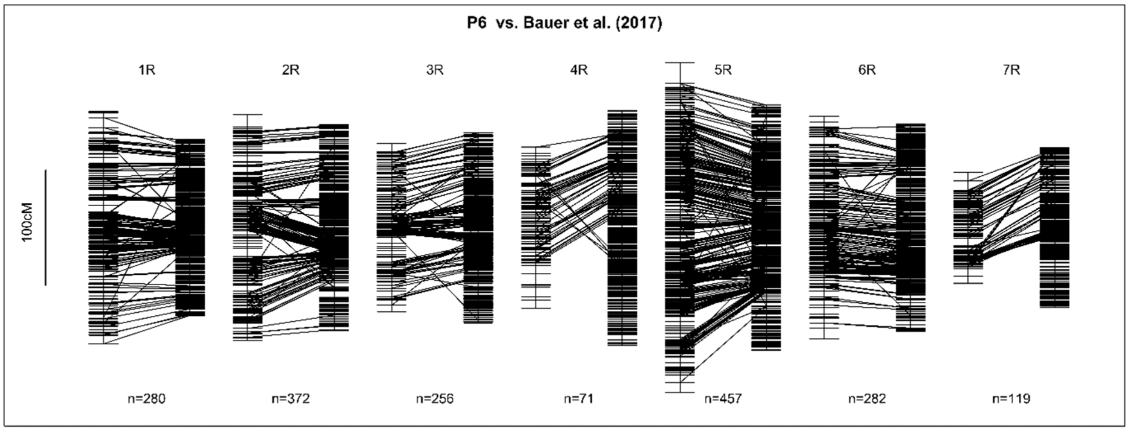

Figure S12 Linkage map comparison (chromosome 1R-7R) of overlapping markers (connected lines) between constructed linkage map based on P6 (left) with the linkage map from Bauer et al. (2017). The link between both maps is based on blasted marker sequences in the rye genome scaffolds of Bauer et al. (2017). The number of common markers/scaffolds (n) is reported.
